# Overcome the challenge for intratumoral injection of STING agonist for pancreatic cancer by systemic administration

**DOI:** 10.1186/s13045-024-01576-z

**Published:** 2024-08-07

**Authors:** Keyu Li, Junke Wang, Rui Zhang, Jiawei Zhou, Birginia Espinoza, Nan Niu, Jianxin Wang, Noelle Jurcak, Noah Rozich, Arsen Osipov, MacKenzie Henderson, Vanessa Funes, Melissa Lyman, Alex B. Blair, Brian Herbst, Mengni He, Jialong Yuan, Diego Trafton, Chunhui Yuan, Michael Wichroski, Xubao Liu, Juan Fu, Lei Zheng

**Affiliations:** 1 Division of Pancreatic Surgery, Department of General Surgery, West China Hospital, Sichuan University, Chengdu, Sichuan 610041 China; 2 Division of Biliary Surgery, Department of General Surgery, West China Hospital, Sichuan University, Chengdu, Sichuan 610041 China; 3 Department of Oncology and The Sidney Kimmel Comprehensive Cancer Center, Johns Hopkins University School of Medicine, Baltimore, MD 21287 USA; 4 The Pancreatic Cancer Precision Medicine Center of Excellence Program, Johns Hopkins University School of Medicine, Baltimore, MD 21287 USA; 5 The Bloomberg Kimmel Institute for Cancer Immunotherapy, Johns Hopkins University School of Medicine, Baltimore, MD 21287 USA; 6 Zhejiang Provisional People’s Hospital, Hangzhou, Zhejiang China; 7 The First-Affiliated Hospital of Zhejiang University, Hangzhou, Zhejiang China; 8 Lake Erie College of Osteopathic Medicine, Erie, PA 16509 USA; 9 Department of Surgery, Johns Hopkins University School of Medicine, Baltimore, MD 21287 USA; 10 The Multidisciplinary Gastrointestinal Cancer Laboratories Program, The Sidney Kimmel Comprehensive Cancer Center, Johns Hopkins University School of Medicine, Baltimore, MD 21287 USA; 11 Cedars-Sinai Medical Center, Los Angeles, CA 90048 USA; 12 Department of General Surgery, Peking University Third Hospital, Beijing, 100191 China; 13 Bristol Myers Squibb Co, Princeton, NJ 08648 USA; 14 The Johns Hopkins Kimmel Cancer Center, 1650 Orleans Street, CRB1 Room 351, Baltimore, MD 21231 USA

**Keywords:** Pancreatic ductal adenocarcinoma, Stimulator of interferon genes, Tumor microenvironment, Immune checkpoint inhibitor, Immunotherapy

## Abstract

Due to the challenge for intratumoral administration, innate agonists have not made it beyond preclinical studies for efficacy testing in most tumor types. Pancreatic ductal adenocarcinoma (PDAC) has a hostile tumor microenvironment that renders T cells dysfunctional. Innate agonist treatments may serve as a T cell priming mechanism to sensitize PDACs to anti-PD-1 antibody (a-PD-1) treatment. Using a transplant mouse model with spontaneously formed liver metastasis, a genetically engineered KPC mouse model that spontaneously develops PDAC, and a human patient-derived xenograft model, we compared the antitumor efficacy between intrahepatic/intratumoral and intramuscular systemic administration of BMS-986301, a next-generation STING agonist. Flow cytometry, Nanostring, and cytokine assays were used to evaluate local and systemic immune responses. This study demonstrated that administration of STING agonist systemically via intramuscular injection is equivalent to its intratumoral injection in inducing both effector T cell response and antitumor efficacy. Compared to intratumoral administration, T cell exhaustion and immunosuppressive signals induced by systemic administration were attenuated. Nonetheless, either intratumoral or systemic treatment of STING agonist was associated with increased expression of CTLA-4 on tumor-infiltrating T cells. However, the combination of a-PD-1 and anti-CTLA-4 antibody with systemic STING agonist demonstrated the antitumor efficacy in the KPC mouse spontaneous PDAC model. The mouse pancreatic and liver orthotopic model of human patient-derived xenograft reconstituted with PBMC also showed that antitumor and abscopal effects of both intratumoral and intramuscular STING agonist are equivalent. Taken together, this study supports the clinical development of innate agonists via systemic administration for treating PDAC.

**Supplementary Information:**

The online version contains supplementary material available at 10.1186/s13045-024-01576-z.

To the editor

Despite promising preclinical studies with innate agonists as potential immunotherapeutics or vaccine adjuvants [1], these agents such as STING [2] and NLRP3 [3] agonists have not been tested in most of the tumor types [4] for efficacy due to the difficulties associated with their intratumoral delivery. To overcome this challenge, we tested the systemic delivery of a next-generation CDN-based STING agonist and compared its anti-tumor efficacy and elicited immune responses with the intratumoral (IT) injection of this agent (IT-STING) first in a mouse liver metastasis model [5] of pancreatic ductal adenocarcinoma (PDAC) to resemble IT and intramuscular (IM) injection of STING agonist in metastatic cancer patients (Fig.S1). These mice were also inoculated with subcutaneous (SubQ) tumors to evaluate the abscopal effect. The results supported an abscopal effect from the STING agonist and anti-PD-1 antibody (a-PD-1) combo treatment (IT-combo). IT-combo significantly prolonged survival compared to STING agonist monotherapy (Fig.S1). Subsequent analysis of tumor-infiltrating leucocytes (TILs) demonstrated that IT-combo enhances effector T cells (Teffs) infiltration and CD103^+^dendritic cells (DCs) [6] in both target and non-target liver metastatic lesions (Fig.S2). NanoString assays showed that IT-combo activates pro-inflammatory pathways broadly including those that mediate the inflammasome (Fig.S3) and enhances T cell activation signals (Fig.S4), which is further supported by the robust increasement of the expression of other chemokines such as *Ccl4* and *Cxcl9* (Fig.S5). Additionally, STING agonist may confer an antitumor effect by suppressing CCL17 expression or CCL17-expressing cells and thereby suppressing Treg migration [7] into the tumor microenvironment (TME) (Fig.S5). Moreover, IT-combo enhanced the infiltration and activation of Teffs in the distant SubQ tumors (Fig.S6-S7). Interestingly, mice who received STING agonist intramuscularly in combination with a-PD-1 (IM-combo) reached the longest survival beyond 6 weeks (Fig. 1A-B; Fig.S8A), suggesting that systemic administration of STING agonist is not inferior to intratumoral administration.

**Fig. 1:**
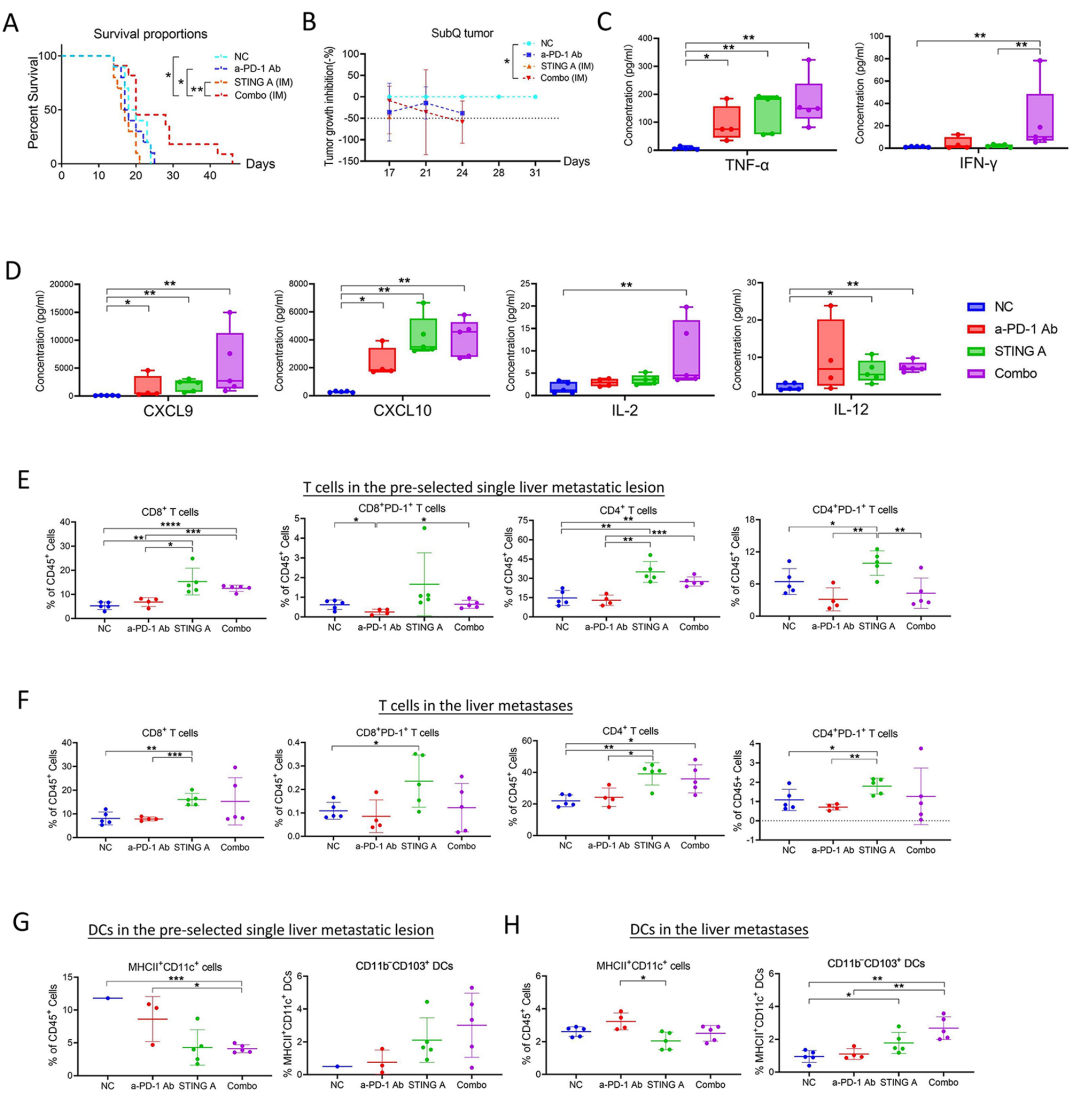
Intramuscular injection of STING agonist in combination with anti-PD-1 antibody prolonged the survival of liver metastasis mice and induces both systemic and intratumoral immune response. Preclinical PDAC metastatic model using Kras/p53/pdx1-Cre (KPC) cells was established in syngeneic C57Bl/6 mice. On day 0, 7.5 × 10^5^ KPC cells were injected by the hemisplenetomy procedure to establish the liver metastatic lesions (Figure S1A). On day 7, 5 × 10^5^ KPC cells were subcutaneously injected into the bilateral flanks of the postoperative mice to establish the SubQ tumors. Mice meeting the prespecified inclusion criteria were randomly assigned to the vehicle and treatment groups: (1) containing at least one target liver metastatic lesion between 3–5 mm in diameter as measured by ultrasonography because target lesions with such a size are easily separated from other metastases and are also larger enough to be feasible for intratumoral (IT) injection; (2) absence of peritoneal implants; (3) presence of palpable bilateral SubQ tumors. STING agonist (BMS986301) was administered intramuscularly (IM) at 5 mg/kg once weekly for three doses, starting on day 14. Anti-PD-1 antibody (BMS936558) was given intraperitoneally twice weekly at 10 mg/kg for five doses, starting on day 14 (Figure S1C). Tumor volumes were measured by ultrasound and tumor growth inhibition (TGI) was calculated using the formula %TGI=(1–[Tt/T0/Ct/C0]/1–[C0/Ct]) ×100, with a TGI > 50% considered significant. (**A**) Kaplan-Meier’s survival curves compare the survival in different IM treatment groups. Systemic administration did not show any obvious toxicity including bleeding, unhealed wound, paralysis, weight loss, etc. All the mice following the hemisplenectomy procedure were candidates for IM injection although only those feasible for IT injection were chosen for the purpose of experimental comparison. Mice in the IM-Combo group had a significantly prolonged survival when compared to other treatment groups. (**B**) TGI on distant SubQ tumors. The dashed line at -50% indicates statistically significant inhibition. The TGI rate for SubQ tumors also exhibited a significant increase in the IM-Combo group (maximum TGI = 58.86 ± 49.12%, *p* < 0.05). Although this study did not demonstrate a significant difference in the treatment response between IM and IT injections of STING agonist, mice who received IM injections of STING agonist in combination with anti-PD-1 antibody survived longer than 6 weeks were observed, whereas none of the mice who received IT injection of STING agonist in combination with anti-PD-1 antibody survived longer than 6 weeks (Figure S8A). Therefore, it might be possible to see the survival benefit of the IM injection of STING agonist if the sample size would potentially be larger; however, the sample size in each experiment was limited by the technical difficulty of IT injection. (**C**) Comparison of serum concentrations of TNF-α and IFN-γ collected 6 h after the first IM injection between treatment groups. The results demonstrated that several cytokines especially those associated with inflammation had a significantly increased level in the sera of mice from the IM-Combo treatment group, including TNF-α and IFN-γ. (**D**) Comparison of serum concentrations of CXCL9, CXCL10, IL-2, and IL-12 collected 6 h after the first IM injection between treatment groups. Interestingly, several T lymphocytes trafficking chemokines including CXCL9 and CXCL10, and type I cytokines including IL-2 and IL-12 were significantly increased in the sera from mice in the combo treatment group compared to other treatment groups, suggesting that IM injection of STING agonist in combination with anti-PD-1 antibody is potentially able to induce an anti-tumor systemic immune response. Percentages of the CD8^+^, CD8^+^PD-1^+^, CD4^+^, and CD4^+^PD-1^+^ T cells among CD45^+^ leucocytes in the pre-selected, target single liver metastatic lesions (**E**) and non-target liver metastases (**F**) are presented. The single target liver lesion was pre-selected as it would be selected for the IT treatment, but without any IT treatment to be given. IM injection of STING agonist alone significantly enhanced the infiltration of CD8^+^ and CD4^+^ T cells in both the pre-selected liver metastatic lesion and other liver metastases. The enhancement of the T cell infiltration was not as high in the IM-Combo group as the IM STING agonist monotherapy group. Percentages of the MHCII^+^CD11c^+^DC and CD11b^−^CD103^+^ subtype among CD45^+^ leucocytes, respectively, in the pre-selected single liver metastatic lesion (**G**) and non-target liver metastases (**H**) are presented. CD103^+^DCs showed a similar profile in the tumors with the IM injection of STING agonist compared to those shown above with the IT injection of STING agonist (Figure S2B and S2D), suggesting that systemic IM injection of STING agonist is able to activate the desired antigen-presenting process in the liver metastases. Taken together, these results suggest that IM injection of STING agonist is able to induce similar systemic immune responses as the IT injection of STING agonist. Systemic IM injection of STING agonist is also able to activate the desired antigen-presenting process in the liver metastases. Although the effector T cell responses appear to be slightly weaker in the IM injection of STING agonist, the increase in the T cell exhaustion and immune checkpoint signals and myeloid cell-recruiting cytokine/chemokine signals that were observed with the IT-Combo treatment (Figure S4D-E) were not observed in the IM-combo group. NC, vehicle/isotype antibody control; STING A, STING agonist; a-PD-1 Ab, anti-PD-1 antibody; IM, intramuscular. Data shown as mean ± SD; comparison by Log-rank test for A and by unpaired t test for others; *p < 0.05; **p < 0.01; ***p < 0.001; ****p < 0.0001

To assess the systemic immune responses induced by IM injection of STING agonist (IM-STING) with or without a-PD-1, we measured the cytokine response in sera as well as intratumoral gene expression. The results suggested that IM-STING agonist induces similar systemic immune responses (Fig. 1C-D; Fig.S8B-C) but attenuated T cell exhaustion and immunosuppressive signals (Fig.S9-S10) compared to NanoString analyses for IT-STING agonist (Fig.S11). Next, we assessed TILs by dissecting a single target liver metastatic lesion and a mixture of non-target liver metastases, respectively, in mice treated with IM-STING agonist. Although the enhancement of T cell infiltration was modestly decreased in the IM-combo group, CD103^+^DCs showed a similar profile in tumors treated with IM-STING agonist (Fig. 1E-H; Fig.S8D-E) compared to IT-STING agonist (Fig.S2), suggesting that systemic STING agonist can activate the desired antigen-presenting process in distant metastases.

We thus validated the antitumor activity of systemic STING agonist in KPC mice that develop invasive PDAC spontaneously [8]. As CTLA-4 remains one of the immunosuppressive signals induced by IM-STING agonist (Fig.S9), we included anti-CTLA-4 antibody in the immune checkpoint inhibitor (ICI) regimen (Fig. 2A). Dual ICIs failed to improve the survival of KPC mice; however, following the co-administration of IM-STING agonist with dual ICIs significantly prolonged the survival (Fig. 2B).

**Fig. 2:**
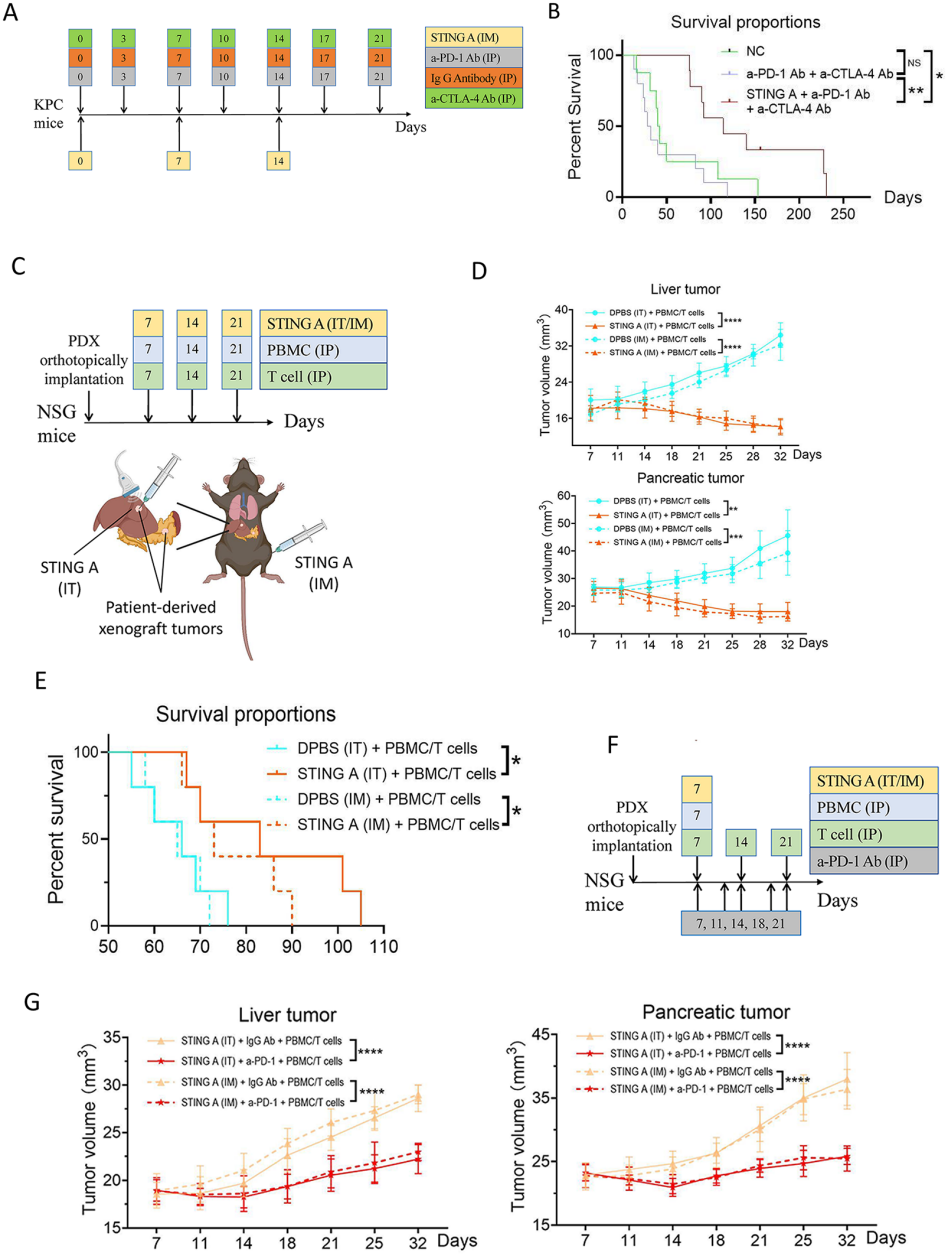
STING agonist exert antitumor efficacy in both the KPC mouse model and the PDX mouse model. (**A**) Treatment schema for genetically engineered KPC mouse model that develops invasive PDAC spontaneously in a manner resembling human PDAC pathogenesis. KPC mice were subjected to weekly to twice weekly ultrasonic screening starting at 3 months of age and enrolled in the experiment randomly once either the length, width, or height of the pancreatic tumor reached 2 mm to ensure that eligible mice had equivalent tumor burdens. As CTLA-4 remains one of the immunosuppressive signals present in tumors treated by the IM STING agonist, the anti-CTLA-4 antibody was included in the immune checkpoint inhibitor regimen. (**B**) Kaplan-Meier survival curves compare overall survival between different treatment groups in the KPC mouse model. Eligible KPC mice were randomized into the three treatment groups to receive a total of seven injections. Treatment toxicity and mouse survival were monitored for 3 months following the first treatment. No treatment related toxicity including local toxicity related to IM injection sites was observed. The STING agonist monotherapy did not show any antitumor activity in a later experiment (manuscript in preparation). Dual checkpoint inhibitors failed to improve the overall survival of KPC mice when compared with control group; however, the co-administration of IM STING agonist with dual checkpoint inhibitors significantly prolonged the survival of the KPC mice. As it would be a challenge to breed a large number of the KPC transgenic mice for being randomized to multiple treatment groups, the combination of anti-PD-1 antibody and anti-CTLA-4 antibody instead of two immune checkpoint inhibitors by itself was tested. Future studies comparing the combination of anti-PD-1 antibody and anti-CTLA-4 antibody with either anti-PD-1 antibody alone or anti-CTLA-4 antibody alone are warranted. (**C**) Schema for the treatment of STING agonist or DPBS control and the reconstitution of ex vivo activated T cells and freshly thawed PBMCs, as indicated, in mice implanted with human PDX. In this PDX model, one piece of tumor was orthotopically implanted in the pancreas of the immunodeficient NOD scid gamma (NSG) mice, then a second piece of the tumor was implanted orthotopically in the liver of the same mice to simulate liver metastasis for IT injection. (**D**) Ultrasound measurement of tumor volumes of the orthotopic liver- and pancreas-implanted tumors treated with STING agonist or DPBS control via IT or IM, as indicated. NSG mice were reconstituted weekly with an ex vivo activated T cell fraction from peripheral blood mononuclear cells (PBMCs) as well as a whole fraction of PBMCs to provide myeloid cells including dendritic cells as described previously for the anti-human PD-1 antibody study and innate agonists on the PDX models, respectively. The implanted primary pancreatic tumors were evaluated for antitumor abscopal effects of IT injection of STING agonist. Tumor volumes in the pancreas and liver were monitored using small-animal ultrasound. The results demonstrated a significant growth suppression on both tumors in pancreas and liver regardless of the routes of administration of STING agonist. (**E**) Kaplan-Meier survival curves of mice with the orthotopic liver- and pancreas-implanted tumors treated with STING agonist or DPBS control via IT or IM, as indicated. IT injection of STING agonist significantly inhibited the growth of implanted tumors in the pancreas, supporting the antitumor abscopal effect of IT injection of STING agonist. These results demonstrated a significant and profound antitumor activity with either IT or IM injection of STING agonist alone in the human PDX model reconstituted with ex vivo activated T cells and the whole PBMC to provide myeloid cells including DCs. It is possible that reconstituted T cells and DCs in the PDX model were in a larger quantity than those infiltrating the tumors in the genetically engineered KPC model or the syngeneic model; therefore, STING agonist became more effective in the PDX model. (**F**) Schema for the treatments of STING agonist alone or in combination with a-PD-1 Ab and the reconstitution of ex vivo activated T cells and freshly thawed PBMCs, as indicated, in mice implanted with human PDX. Considering the potent tumor suppression observed after three doses of STING agonist in NSG mice with reconstituted immune systems, the doses of STING agonist and PBMCs were reduced in subsequent experiments. The modified experimental design involved a single infusion of STING agonist and freshly thawed PBMCs while ex vivo activated T cells were still infused three times, each together with each administration of anti-PD-1 antibody. Here, freshly thawed PBMC was only infused once because the effect of STING agonist would be carried over if PBMC continued to be infused for multiple times. (**G**) Ultrasound measurement of tumor volumes of the orthotopic liver- and pancreas-implanted tumors treated with STING agonist alone via IT or IM, as indicated, or in combination with a-PD-1 Ab. Results indicated that a single injection of STING agonist with freshly thawed PBMC infused once in combination with three weekly treatments of anti-PD-1 antibody together with three weekly infusions of ex vivo activated T cells led to a significant tumor suppression after Day 18, when compared to a single injection of STING agonist. NC, vehicle/isotype antibody control; STING A, STING agonist; a-PD-1 Ab, anti-PD-1 antibody; a-CTLA-4 Ab, anti-CTLA-4 antibody; PBMC, peripheral blood mononuclear cell; PDX, patient-derived xenograft; IT, intratumoral; IM, intramuscular; IP, intra-peritoneal. Data shown as mean ± SD; comparison by two-way ANOVA and Log-rank test; *p < 0.05; **p < 0.01; ***p < 0.001; ****p < 0.0001; NS, not significant

To further examine the human patient relevance, we employed a human PDX model of PDAC utilized previously [9, 10], reconstituted weekly (Fig. 2C) with an ex vivo activated T cell fraction from PBMCs as well as a whole fraction of PBMCs to provide myeloid cells including dendritic cells as described previously for the anti-human PD-1 antibody study and innate agonists on the PDX models, respectively [11, 12]. The results demonstrated significant growth suppression in both pancreatic and liver tumors, regardless of the route of administration of the STING agonist (Fig. 2D). Additionally, mice receiving either IT or IM injections of STING agonist had significantly longer survival than their corresponding control groups (Fig. 2E). A single injection of STING agonist with PBMC infusion plus three weekly treatments of a-PD-1 together with infusions of T cells led to a significant tumor suppression after Day 18, when compared to a single injection of STING agonist (Fig. 2F-G). Furthermore, IM-STING agonist resulted in an increased general Teff infiltration whereas IT-STING agonist resulted in a decrease of the cytotoxic Teff subset (Fig.S12). Taken together, our studies suggested that IM-STING agonist yields an antitumor efficacy comparable to IT-STING agonist in the PDX model resembling human liver-metastatic PDACs. Our study also supported the feasibility of administrating an NLRP3 agonist systemically (Fig.S13) and would support a new paradigm of the clinical development of innate immune agonists by systemic administration. This study has thus supported the phase-1 trial evaluating BMS-986301 intratumoral or intravenous injection as monotherapy or in combination with nivolumab/ipilimumab in solid tumors (NCT03956680).

### Electronic supplementary material

Below is the link to the electronic supplementary material.

Supplementary Material 1

## Data Availability

All data needed to evaluate the conclusions in the paper are present in the paper and the Supplementary Materials. Any further information required to support our data will be supplied upon request.

## Abbreviations

PDAC: Pancreatic ductal adenocarcinoma
TME: Tumor microenvironment
STING: Stimulator of interferon genes
PD-1: Programmed cell death protein 1
CTLA-4: Cytotoxic T-lymphocyte associated protein 4
KPC: Kras/p53/pdx1-Cre
NLRP3: NACHT, LRR, and PYD domains-containing protein 3
IFN: Interferon; CDN, cyclic dinucleotide
ICI: Immune checkpoint inhibitors
TGI: Tumor growth inhibition
TIL: Tumor infiltrating leucocyte
DC: Dendritic cell; IL, interleukin
CXCL: C-X-C motif chemokine ligand
CXCR: C-X-C motif chemokine receptor
CCL: C-C motif chemokine ligand
G-CSF: Granulocyte colony stimulating factor
CCR: C-C motif chemokine receptor
PDX: Patient-derived xenograft
PBMC: Peripheral blood mononuclear cell
