# Supplementary material for: Overcome the challenge for intratumoral injection of STING agonist for pancreatic cancer by systemic administration
Source: J Hematol Oncol. 2024 Aug 7;17:62. doi: 10.1186/s13045-024-01576-z (PMC11305077; doi:10.1186/s13045-024-01576-z)
Supplement: Supplementary file 1 — Supplementary Material 1 [file 13045_2024_1576_MOESM1_ESM.docx]

**Supplementary Materials**

**Contents**

**Methods**

**Supplementary figures and legends**

**Figure S1.** Develop a mouse model to resemble the intratumoral injection of STING agonist in metastatic cancer patients showing that the combination of STING agonist and anti-PD-1 antibody significantly prolonged survival comparing to the control treatment and the STING agonist treatment.

**Figure S2.** Intratumoral injection of STING agonist in combination with anti-PD-1 antibody enhances effector T cells and CD103^+^ dendritic cell infiltration in both target liver metastatic lesions and non-target metastatic lesions.

**Figure S3.** Intratumoral injection of STING agonist in combination with anti-PD-1 antibody activate anti-tumor immunity via innate immune response signaling pathways.

**Figure S4.** Intratumoral injection of STING agonist in combination with anti-PD-1 antibody enhances T cell activation signals and reveals differential expression of effector and regulatory genes.

**Figure S5.** Differential expression analysis of cytokines and chemokines in liver metastatic lesions following intratumoral STING agonist and anti-PD-1 antibody treatment.

**Figure S6.** Intratumoral injection of STING agonist in combination with anti-PD-1 antibody enhances effector T cell infiltration status and activation signals in distant subcutaneous tumors, mediating abscopal effects.

**Figure S7.** Gene expression analysis reveals enhanced interleukin and chemokine signaling in distant subcutaneous tumors following intratumoral combination treatment.

**Figure S8.** Intramuscular injection of STING agonist combined with anti-PD-1 antibody induces enhanced serum cytokine levels and tumor-infiltrating immune cells.

**Figure S9.** Gene expression analysis of liver metastatic lesions after STING agonist systemic injection reveals moderate immune modulation but reduced immunosuppressive signals.

**Figure S10.** Analysis of immune cell infiltration and gene expression in SubQ tumors of IM-treated mice reveals modulation of immune responses and checkpoint signaling.

**Figure S11.** Comparison of expressions of differentially expressed genes in tumors from systemically treated mice.

**Figure S12.** Immune modulating effects of STING agonist in combination with anti-PD-1 antibody in the PDX model reconstituted with human PBMC.

**Figure S13.** Systemic administration and intratumoral administration of NLRP3 agonist in combination with anti-PD-1 antibody induced antitumor efficacy.

**Methods**

**Cell lines and media**

The KPC (*LSL-Kras^G12D/+^*; *LSL-Trp53^R172H/+^*; *Pdx-1-Cre*) tumor cell line is a PDAC cell line that was previously established from a C57Bl/6 background mouse model as previously described[1]. The KPC cells were cultured at a temperature of 37°C in a 5% CO_2_ incubator using RPMI 1640 media (Life Technologies). The media was supplemented with 10% heat-inactivated fetal bovine serum (HI-FBS, Benchmark), 1% penicillin/streptomycin (pen/strep, Life Technologies), 1% MEM Non-Essential Amino Acids Solution (MEM-NEAA, Life Technologies), 1% L-glutamine (Life Technologies), and 1% sodium pyruvate (Sigma). Harvested tumor-infiltrating immune cells were processed using T cell culture media consisting of RPMI 1640 media (Life Technologies) supplemented with 10% heat-inactivated fetal bovine serum (HI-FBS, Benchmark), 1% penicillin/streptomycin (pen/strep, Life Technologies), 1% MEM Non-Essential Amino Acids Solution (MEM-NEAA, Life Technologies), 1% HEPES (Life Technologies), 1% L-glutamine (Life Technologies), and 0.05% 2-mercaptoethanol (Sigma).

**Murine models and in vivo experiments**

The in vivo study was conducted following the guidelines established by the Animal Care and Use Committee of Johns Hopkins University. Female C57Bl/6 mice aged 6 to 8 weeks and both male and female NSG mice at the age of 8 to 10 weeks were purchased from Jackson Laboratories and maintained under the Institutional Animal Care and Use Committee (IACUC) guidelines. Third-party management was responsible for maintaining the IACUC mouse protocol.

**Liver metastasis model:**

The hemi-spleen preclinical pancreatic cancer metastatic model using KPC cells was performed on the C57Bl/6 mice as described previously[2]. Briefly, on day 0, the spleen was eviscerated from the anesthetized mouse, clipped and divided in half. One-half of the spleen was injected with 7.5 × 10^5^ KPC cells resuspended in 100 μL of PBS with the anti-clumping agent (Life Technologies) at a dilution of 1:1000 and flushed with 150 μL of PBS in the same syringe. The injected hemi-spleen segment was then surgically removed to eliminate residual tumor cells. The KPC cells were passaged and cultured until day 7, on which 5 × 10^5^ cells were subcutaneously injected into the bilateral flanks of the postoperative mice. Small-animal ultrasound (Vevo770, VisualSonics) was employed to screen for liver metastatic lesion on day 12. Mice that met the prespecified inclusion criteria, as follows, were randomly assigned to the vehicle and treatment groups: 1) containing at least one target liver metastatic lesion between 3 to 5 mm in diameter as measured by ultrasonography because target lesions with such a size are easily separated from other metastases and are also larger enough to be feasible for intratumoral injection; 2) absence of peritoneal implants; 3) presence of palpable bilateral SubQ tumors.

Stimulator of interferon genes (STING) is a transmembrane protein that is expressed in various endothelial, epithelial and hematopoietic cells. Upon activation at the endoplasmic reticulum and subsequent translocation to the Golgi, STING recruits and activates TANK-binding kinase 1 (TBK1), which in turn phosphorylates and activates interferon regulatory factor 3 (IRF3) and NF-kB transcriptional programs, resulting in the expression and release of pro-inflammatory type I interferons (IFNs) and cytokines[3-5].

The NACHT, LRR, and PYD domains-containing protein 3 (NLRP3) inflammasome as a family member of the NOD-like receptors, is a critical component of the innate immune system, sensing cellular perturbations and microbial threats to initiate inflammatory responses. Activation of NLRP3 leads to assembly of inflammasomes, triggering pyroptosis and release of proinflammatory cytokines, such as IL-1β and IL-18[6]. This pathway plays a pivotal role in inhibiting neoplastic events, potentially through induction of IFN-γ production and STAT1 signaling.

**Dosing of innate agonists:**

Accumulating evidence has suggested that STING also possesses cell-intrinsic tumor suppressive activity[7]. The mechanistic underpinnings of the cyclic GMP-AMP synthase-STING pathway make STING agonists a promising adjuvant to cancer vaccines[8]. Numerous natural or synthetic STING agonists as monotherapy or in combined with other treatments have also been tested in both pre-clinical studies and clinical trials across many cancer types[9, 10]. Synthetic cyclic dinucleotides (CDNs) were the first generation of STING agonists that entered the clinical trial phase of drug development due to their structural versatility and ability to bind all prevalent allelic variants of human STING. BMS-986301 is a novel CDN-based, next-generation STING agonist and has demonstrated a promising antitumor activity in the CT26 and MC38 subcutaneous tumor murine models, resulting in more than 90% of tumor regression compared to only 13% with the first-generation STING agonist, ADU-S100. Similar results were observed with a single intratumoral administration of BMS-986301 in combination with an anti-PD-1 agent [11]. However, these previous studies were limited by the subcutaneously implanted tumors, which do not resemble the TME of human PDAC[12]. In liver metastasis model, the STING agonist (BMS986301) was dissolved into DPBS (Life Technologies) vehicle and administered to tumor-bearing mice either by intratumoral (IT) or intramuscular (IM) injection once a week at a dose of 5 mg/kg starting on day 14, for a total of three doses. An anti-mouse PD-1 antibody (BMS936558, BMS) and an IgG control (ab18443, Bristol-Myers Squibb) were administered intraperitoneally twice weekly starting on day 14, for a total of five doses, at a dose of 10 mg/kg.

BMS-986299, an imidazole-fused 2-aminoquinoline NLRP3 agonist[13], has been shown to stimulate the production of proinflammatory cytokines IL-1β and IL-8. Its potential immunomodulatory and antineoplastic activities are currently under investigation. In this study, the NLRP3 agonist BMS-986299 was dissolved in DPBS (Life Technologies) vehicle and administered via intramuscular, intravenous, or intra-subcutaneous tumor injection once a week for three consecutive weeks. The dose of BMS-986299 was 5 mg/kg, starting on day 14, with a total of three doses administered.

**Orthotopic PDX model:**

The orthotopic mouse model was established on the immunodeficient NSG mice. First, the cryopreserved JH029 PDX tumors[14] were recovered and subcutaneously inoculated on the bilateral flanks of the NSG mice. After 2-3 weeks, the growing SubQ tumors were harvested and dissected into cubes with a diameter of approximately 2 millimeters. The tumor cubes were then orthotopically implanted into the pancreas[15] and liver[16] of the new NSG mice as previously described. In brief, one piece of tumor was implanted approximately 10 millimeters above the lower edge of the left lobe of the liver where it was optimal for ultrasound tracking and ultrasound-guided injections. A second piece of tumor was implanted into the pancreas of the same mice during the same procedure. Seven days following the tumor implantation, the NSG mice were randomized into different treatment groups as indicated.

Peripheral blood mononuclear cells (PBMCs) were harvested from healthy donors’ peripheral blood by Ficoll–Paque (GE Healthcare) gradient centrifugation. PBMCs were cultured in the medium containing RPMI 1640 (Life Technologies), 10% FBS (Benchmark), 100 U/ml pen/strep (Life Technologies), and 30 U/ml recombinant IL-2 (Millipore Sigma). The PBMC culture was stimulated by the Human T-Activator CD3/CD28 Dynabeads™ (Thermo Fisher Scientific) for T cell expansion and activation as per the manufacturer’s protocol, followed by incubation with it at 37°C and 5% CO_2_ for 5-6 days before the T cells were harvested for injection.

Starting on Day 7 following tumor implantation, STING agonist (BMS986301) dissolved in the DPBS (Life Technologies) vehicle was administered via IT or IM injection as described above at a dose of 5 mg/kg once a week for once or three weekly times as indicated. Immediately following the administration of the STING agonist, 1 × 10^6^ activated T cells freshly harvested from the above culture and 1 × 10^6^ freshly thawed PBMCs that provide myeloid cells including dendritic cells were infused into the mice via intraperitoneal injection, as described previously for the anti-human PD-1 antibody study and innate agonists on the PDX models, respectively[17, 18]. T cells were infused once a week for three weekly times, and freshly thawed PBMCs were infused once a week for once or three weekly times as indicated.

**Tumor measurement:**

All the mice were monitored until death, with survival monitoring of NSG mice for 80 days. If any of the following survival endpoints were met: hunchback posture, lethargy, dehydration, and rough hair coat, euthanasia was performed via CO_2_ inhalation. Tumor volumes of liver and pancreatic tumors in all mouse models including the KPC transgenic mice and NSG mice was measured using small-animal ultrasound, while SubQ tumors were measured with calipers. TGI of the liver metastatic lesion and SubQ tumors was calculated during the dosing period using the formula: %TGI=(1–[Tt/T0/Ct/C0]/1–[C0/Ct]) ×100, where Tt is the median tumor volume of the treated group at time t, T0 is the median tumor volume of the treated group at time 0, Ct is the median tumor volume of the control group at time t and C0 is the median tumor volume of the control group at time 0. A TGI of greater than 50% is considered meaningful.

**Cell staining and flow cytometry**

For the KPC syngeneic liver metastasis model, on Day 21, various samples were collected from mice that received intratumoral injections of STING agonist, including the specific liver metastatic lesion received dosing injection, the whole left liver (including other metastatic lesions), and one of the SubQ tumors. Tumor-infiltrating immune cells were analyzed from these samples. First, the samples were mechanically minced using the gentleMACS Dissociator with Tumor Dissociation Kit for mouse tissues (Miltenyi Biotec, San Diego, CA, USA) and processed as previously described to collect the lymphocytes[19]. Then, leukocytes were isolated from the liver and tumor samples and stained with the Live Dead Aqua Dead Cell Kit (Invitrogen). The leukocytes were washed and blocked using mouse Fc antibody (BD Pharmingen) for 10 minutes on ice. Afterwards, cell-surface antibodies were used to stain the cells, including CD45-PerCP-Cy5.5 (Biolegend), CD4-APC-A750 (Biolegend), CD8a-PE/Cy7 (Biolegend), PD-1–FITC (Biolegend), CD45-APC-Cy7 (Biolegend), CD11b-PE TexasRed (Invitrogen), MHC II (I-A, I-E)- FITC (Biolegend), CD11c-APC (Biolegend), F4/80-PE/Cy7 (Invitrogen), CD8a-V450 (Biolegend), and CD103-PE (Biolegend), for a 30-minute incubation on ice. The cells were then washed twice and resuspended in FACS buffer, and flow cytometry was performed using CytoFLEX (Beckman Coulter). Flow data were analyzed using the CytExpert software (Beckman Coulter).

For the orthotopic PDX model, on Day 24, liver and pancreatic tumors were collected and processed as above described. Tumor-infiltrating immune cells were isolated from these samples. Cell-surface antibodies were used to stain the cells, including CD45-PE/Cy5 (Biolegend), CD3-FITC (Biolegend), CD4-BV650 (Biolegend), CD8-BV421 (Biolegend), FOXP3-PE (Biolegend), IFNγ-APC (Biolegend), MHC II-PE CF594 (Biolegend), CD11b-BV605 (Biolegend), CD103-BV711 (Biolegend), and CD86-PerCP-Cy5.5 (Biolegend), followed by flow cytometry analysis.

**Luminex Based Cytokine Profiling**

For serum cytokine detection analyses, peripheral blood was collected from submandibular facial vein on day 14 six hours after the first treatment. After collection, the blood was immediately centrifuged at 10 000 g and the resulting serum was collected. A total of 44 individual analytes were included in this study, which were divided into three panels (cat. MCYTMAG-70K-PX32, MHSTCMAG-70K, and MTH17MAG-47K). Mouse-specific Luminex reagents were used for the analysis, sourced from Millipore Sigma. The cytokine measurements were performed using protocols provided by the manufacturer, which were miniaturized to fit the 384-well format (REF)[20]. The lyophilized cytokine standard cocktails (Millipore Sigma) were prepared according to the manufacturer's instructions. 30 μL of mouse serum was submitted for profiling in the 96-well format at −70℃. The samples were then thawed on ice and diluted 1:1 in the Luminex kit assay buffer. Next, 10 μL of the diluted serum, standard, and kit-provided control samples were transferred to 384-well assay plates (Greiner, cat. 781096) and mixed with 10 μL Luminex bead reagents. The plates were shaken overnight at 4℃ in the dark and washed twice with 90 μL of 1X wash buffer using a magnetic plate washer (Biotek). Then, 10 μL of the detection antibody solution was added to each well and mixed. The plates were shaken for 1 hour at room temperature in the dark. After adding 10 μL of Streptavidin labeled Phycoerythrin to each well, the assay plates were incubated for 30 min at room temperature in the dark. Following the incubation period, the plates with 90 μL of 1X wash buffer, and 80 μL of sheath fluid were added to each well. The plates were then measured using the Bio-Plex 3D system (Bio-Rad), and the data were analyzed using the Bio-Plex Software 3.0. A total of 70 μL and a minimum of 50 bead events per analyte were acquired. The mean fluorescence intensity data were converted into pg/mL using a 4-parametric logistic fit model based on the standard curve.

**NanoString**

After the mouse was euthanized and the tumor was harvested, the tumor tissues were submerged into RNA-later (Invitrogen) to preserve the RNA. The total RNA was extracted from the whole specimen using the AllPrep DNA/RNA/Protein Mini Kit (Qiagen) according to the manufacturer's instructions. The RNA was then equalized for NanoString hybridization using the Formulatrix Tempest. The murine PanCancer Immune panel codeset (XT_PGX_MmV1_CancerImm_CSO, cat. 115000142), which contains 750 target genes along with housekeeping and negative/positive control probes, was used for the NanoString hybridization. The data obtained was then analyzed using NanoString nSolver 3.0 and an internally developed NanoString Data Analyzer Rshiny app (BMS). The samples were run on the NanoString MAX system reader, and the data was analyzed using various CRAN and Bioconductor packages such as dplyr, tidyr, and reshape2 to clean, reformat and match the sample annotations to the normalized data exported from NanoString nSolver V.3.0.

**Statistical analyses**

Statistical analyses and graphing were conducted using GraphPad Prism software (GraphPad Software). The tumor inhibition rates were analyzed using the unpaired t-test while Kaplan-Meier curves and log-rank tests were utilized to evaluate the survival outcomes across the groups. The unpaired t-test was used to determine the mean values for cell number, cytokine expression, and NanoString data. A p-value <0.05 was considered statistically significant.

Supplementary Figures


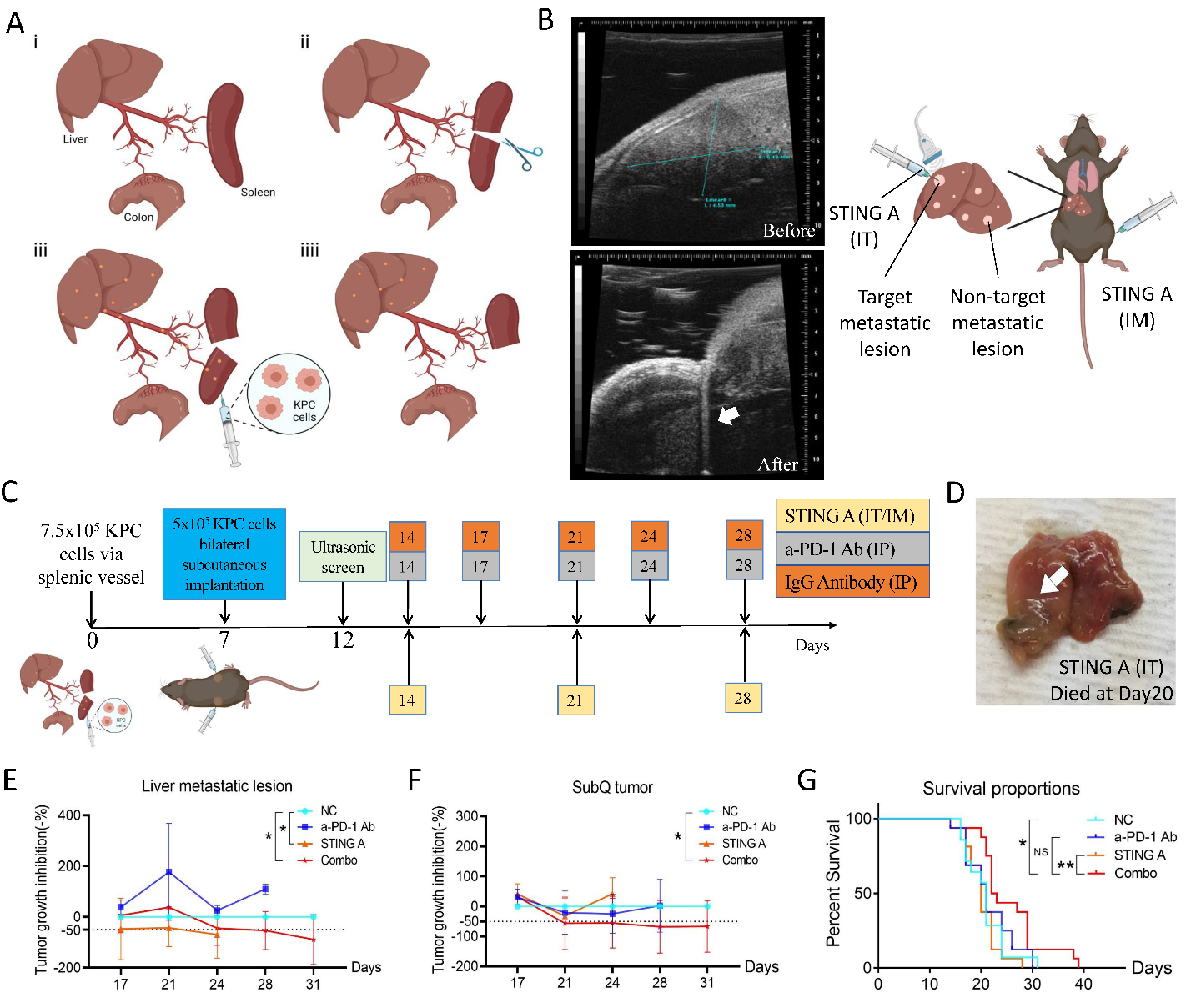


**Figure S1. Develop a mouse model to resemble the intratumoral injection of STING agonist in metastatic cancer patients showing that the combination of STING agonist and anti-PD-1 antibody significantly prolonged survival comparing to the control treatment and the STING agonist treatment. (A)** The schema of the hemisplenectomy procedure and splenic vessel injection to form liver metastases. To examine whether systemic administration of STING agonist is equivalent to intratumoral (IT) injection, two mouse models were established to compare these two routes of innate agonist administration. Firstly, a model resembling the IT injection of STING agonists in patients with metastatic diseases was developed. This involved the implantation of 7.5 x 10^5^ tumor cells of the KPC tumor cell line, derived from the PDAC of the Kras/p53/pdx1-Cre (KPC) mouse model, via the hemisplenectomy procedure and splenic vessel injection to form liver metastases. The mice in this model would uniformly die from liver metastases if not treated[2]. **(B)** Ultrasonographic measurement for the target liver metastatic lesion before and after intratumoral injection of the STING agonist. Arrow indicates the injection needle path. The changes in the sizes of the metastatic lesions in the liver can be monitored by small-animal ultrasonography. Liver metastases have been identified as potential sites for the IT administration of the first-generation STING agonists to treat patients with PDAC. In this study, the liver metastases serve as the clinically relevant target tumors for IT administration of STING agonist. **(C)** Treatment schema. Mice were also inoculated subcutaneously with the KPC tumor cell line to form subcutaneous (SubQ) tumors, not for the IT injection of STING agonist, but for the abscopal effect to be examined. Mice that met inclusion criteria were randomly assigned to each group on day 12 and monitored twice a week until survival endpoints were met. Sixteen mice per group were treated by control vehicles (negative control, NC), anti-PD-1 antibody (a-PD-1 Ab), STING agonist (STING A), and STING agonist given in combination with anti-PD-1 antibody (Combo). A total of five doses of anti-PD-1 antibody was administered twice a week by intraperitoneal injection (IP), while the STING agonist was given for three weekly doses by IT injection to mimic local treatment or intramuscular (IM) injection to mimic systemic treatment. It should be noted that only approximately 35% of mice in the hemisplenectomy model harbored a liver metastasis feasible for IT injection. Mouse survival was followed; the target liver metastatic lesion was measured by ultrasound; and the subQ tumors was measured by calipers twice a week. **(D)** No obvious signs of toxicity, such as bleeding, infection, paralysis, or weight loss, were observed following the IT injection of the STING agonist. However, small areas of liver necrosis were noticed around the injection sites during the necropsy of two mice. A representative liver displaying this effect is presented. **(E)** Tumor Growth Inhibition (TGI) of the injected liver metastatic lesion during the treatment period. Dashed line at -50% indicates statistically significant TGI. The results showed that the TGI rate of the target liver metastasis lesion was significantly increased in the STING agonist and anti-PD-1 antibody combination treatment group (maximum TGI=88.68±98.42%, p <0.05) and the STING agonist group (maximum TGI=69.38±73.94%, p <0.05) as compared to the negative vehicle group. **(F)** To investigate whether the local intratumoral injection of STING agonist could induce the abscopal effect, subcutaneous tumors were implanted on the bilateral flanks of the liver metastasis mice model to mimic distant metastases. The TGI rate of bilateral SubQ tumors was shown, which is also significantly increased in the combo group (maximum TGI=66.38±86.00%, p <0.05) as compared with negative control. Interestingly, the TGI in the distant SubQ tumors is bigger than that of the locally targeted liver metastatic lesion, supporting an abscopal effect from the STING agonist and anti-PD-1 antibody combo treatment. **(G)** Kaplan-Meier survival curves compare different treatment groups. Analysis revealed that the combo treatment significantly prolonged survival comparing to the control treatment and the STING agonist treatment. Nevertheless, the combo treatment prolonged survival modestly without a statistical significance comparing to the anti-PD-1 antibody treatment. It is possible that the local intratumoral injection of saline may have caused inflammatory response which resulted in a small effect on priming the tumor for the anti-PD-1 antibody treatment. Nevertheless, the combo treatment significantly prolonged survival comparing to the control treatment and the STING agonist treatment. NC, vehicle/isotype antibody control; STING A, STING agonist; a-PD-1 Ab, anti-PD-1 antibody; Combo, STING A+a-PD-1 Ab; IT, intratumoral; IM, intramuscular; IP, intra-peritoneal. Data shown as mean ± SD; comparison by unpaired t test in E and F, and by Log-rank test in G; *p < 0.05; **p < 0.01; NS, not significant.


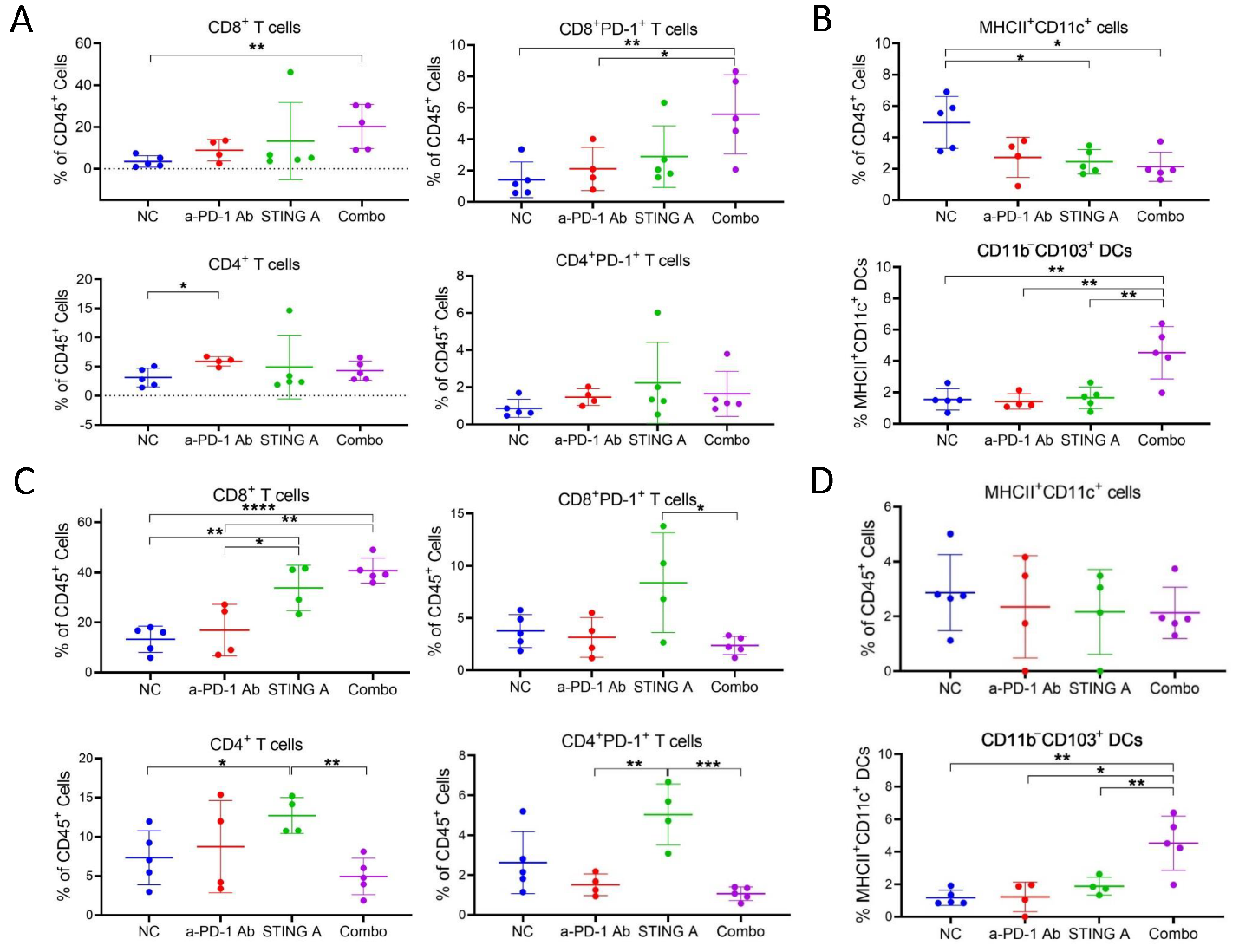


**Figure S2. Intratumoral injection of STING agonist in combination with anti-PD-1 antibody enhances effector T cells and CD103^+^ dendritic cell (DC) infiltration in both target liver metastatic lesions and non-target metastatic lesions.** To understand the mechanistic basis of the enhanced antitumor activity of STING agonist in combination with anti-PD-1 antibody, flow cytometry analysis of the tumor infiltrating leucocytes (TILs) derived from the targeted liver metastasis in the same PDAC mouse model was conducted. As described in the dosing schema in Figure S1C, mice received the anti-PD-1 antibody on days 14 and 17, and the STING agonist on days 14. On day 21, the mice were sacrificed and the implanted SubQ tumors as well as livers were harvested. TILs from the targeted liver metastatic lesion and the whole liver tissue excluding the targeted lesion were compared. Note that the whole liver tissue harvested on day 21 was diffusely infiltrated by non-target metastatic lesions. **(A)** Percentages of the CD8^+^, CD8^+^PD-1^+^, CD4^+^, CD4^+^PD-1^+^ T cells among CD45^+^ leucocytes in the target liver metastatic lesion. The results showed that the STING agonist + anti-PD-1 antibody combo therapy significantly increased the infiltration of the CD8^+^ and CD8^+^PD-1^+^ T cells, but not the CD4^+^ and CD4^+^PD-1^+^ T cells in the target lesion comparing to treatment controls. **(B)** Percentages of the MHCII^+^CD11c^+^ DC among CD45^+^ leucocytes and the percent of CD11b^−^CD103^+^ DC subtype in the target liver metastatic lesion. Interestingly, this result indicated that anti-PD-1 antibody, STING agonist, or their combo all decreased the MHCII^+^CD11c^+^ DCs; however, comparing to the treatment control, anti-PD-1 antibody alone, or STING agonist alone, the anti-PD-1 antibody and STING agonist combo significantly increased the CD11b^−^CD103^+^ subtype of DCs, which are known to play a role in the cross-presentation of tumor antigens[21]. **(C)** Percentages of the CD8^+^, CD8^+^PD-1^+^, CD4^+^, CD4^+^PD-1^+^ T cells among CD45^+^ leucocytes in the non-target liver metastases. TILs from non-target liver metastases showed that the combo treatment resulted in a significant increase in CD8^+^ T cells comparing not only to the treatment control, but also to anti-PD-1 antibody alone. The infiltration of CD8^+^PD-1^+^ T cells in the combo group was comparable with the control group and significantly lower than that in the STING agonist alone group. Moreover, CD4^+^ T cells and CD4^+^PD-1^+^ T cells were both significantly decreased in the combo treatment group compared to the STING agonist treatment group, presumably due to the treatment effect of anti-PD-1 antibody. These results suggest that, in non-target liver metastatic lesions, CD8^+^ and CD4^+^ T cells both trended in the favor of antitumor immune response following the anti-PD-1 antibody and STING agonist combo treatment. **(D)** Percentages of MHCII^+^CD11c^+^ DC among CD45^+^ leucocytes and the percent of CD11b^−^CD103^+^ DC subtype in the non-target liver metastases. In non-target liver metastases, MHCII^+^CD11c^+^ DCs were similar among all treatment groups, suggesting that an enhanced antigen presentation was originated in locally targeted lesions. However, CD11b^−^CD103^+^ DCs were significantly elevated in the combo treatment group in non-target lesions. It is possible that CD103^+^ DCs trafficked from targeted lesions to non-target lesions. Taken together, these results suggest that both STING agonist and anti-PD-1 antibody are required to activate local immune response in favor of antitumor response and that this immune response is extended to the non-target lesions in the vicinity of the target lesion. Data are shown as the mean ± SD; comparison by unpaired t test; *p < 0.05; **p < 0.01; ***p < 0.001; ****p < 0.0001.


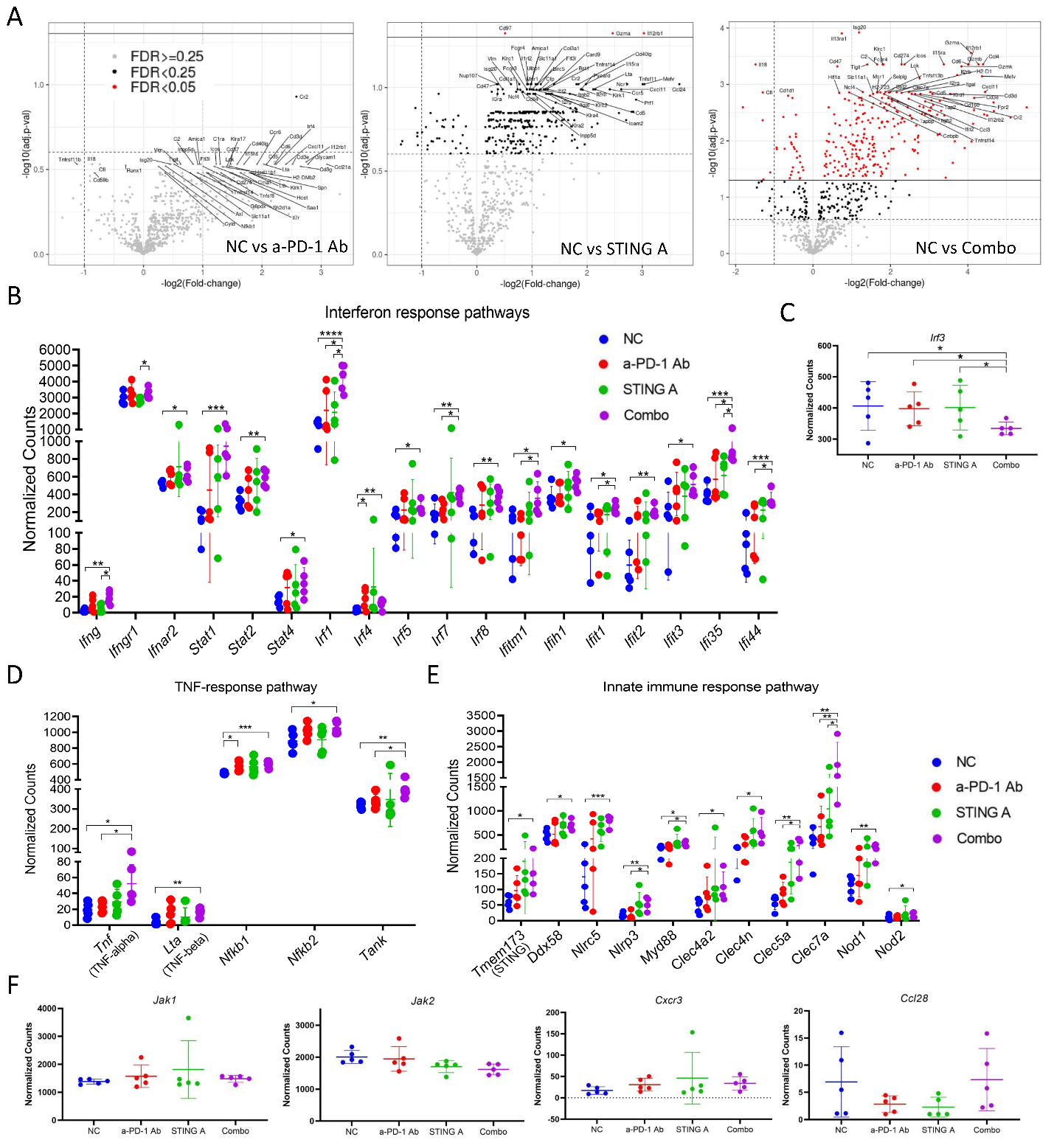


**Figure S3. Intratumoral injection of STING agonist in combination with anti-PD-1 antibody activate anti-tumor immunity via innate immune response signaling pathways. (A)** Volcano map of differentially expressed genes in target liver metastatic lesion between the vehicle control and three treatment groups, respectively. Dashed horizontal line indicates the false discovery rate at 0.25 while the solid horizontal line is at 0.05. Dashed vertical line at -1 indicates the –log2(Fold-change). As flow cytometry analysis has limitations in examining cytokines/chemokines and intracellular signals involved in the innate immune response, the gene regulation of the innate immune response following STING agonist and/or anti-PD-1 antibody treatment was assessed here using NanoString assays with the murine PanCancer Immune panel. Differentially expressed genes were selected by comparing the combo treatment of STING agonist and anti-PD-1 antibody with treatment controls, STING agonist alone, or anti-PD-1 antibody alone, with a 5% false discovery rate. **(B)** Expression of genes in the interferon (IFN)-response pathways in the target liver metastatic lesions from different treatment groups. The gene expression in the IFN response pathways was firstly assessed. Significant upregulation of IFN-γ (*Ifng*) gene expression was observed in the combo treatment group, accompanied by significantly increased expression of *Ifngr1*, *Ifnar2*, *Irf1*, *Irf4*, *Irf5*, *Irf7*, *Irf8*, *Stat1*, *Stat2*, *Stat4*, *Ifitm1*, *Ifih1*, *Ifit1*, *Ifit2*, *Ifit3*, *Ifi35*, and *Ifi44* compared to the control or single treatment groups. **(C)** Expression of *Irf3* in the target liver metastatic lesions from different treatment groups. However, *Irf3* was significantly decreased in the combo treatment group compared to the anti-PD-1 antibody treatment group. **(D)** Expression of genes in the tumor necrosis factor (TNF)-response pathways in the target liver metastatic lesions from different treatment groups. The TNF-response pathway genes including *Tnf* (encoding TNF-α), *Lta* (encoding TNF-β), *Nfkb1, Nfkb2*, and *Tank* were also significantly upregulated in the combo treatment group compared to the control or single treatment groups. **(E)** Expression of genes in the innate immune response pathways in the target liver metastatic lesions from different treatment groups. The innate immune response pathways genes including *Tmem173* (*STING*), *Ddx58*, *Nlrp3*, *Nlrc5*, *Myd88*, *Clec4a2*, *Clec4n*, *Clec5a*, *Clec7a*, *Nod1*, and *Nod2* were significantly increased in the combo treatment group compared to the control or single treatment groups. **(F)** Expression of *Jak1*, *Jak2*, *Cxcr3*, and *Cd28* in the target liver metastatic lesions from different treatment groups. However, as anticipated, the gene expression of *Jak1* and *Jak2* was similar among all treatment groups, suggesting *Jak1/2* are not regulated at the RNA level. Data are shown as the mean ± SD; comparison by unpaired t test; *p < 0.05; **p < 0.01; ***p < 0.001; ****p < 0.0001.


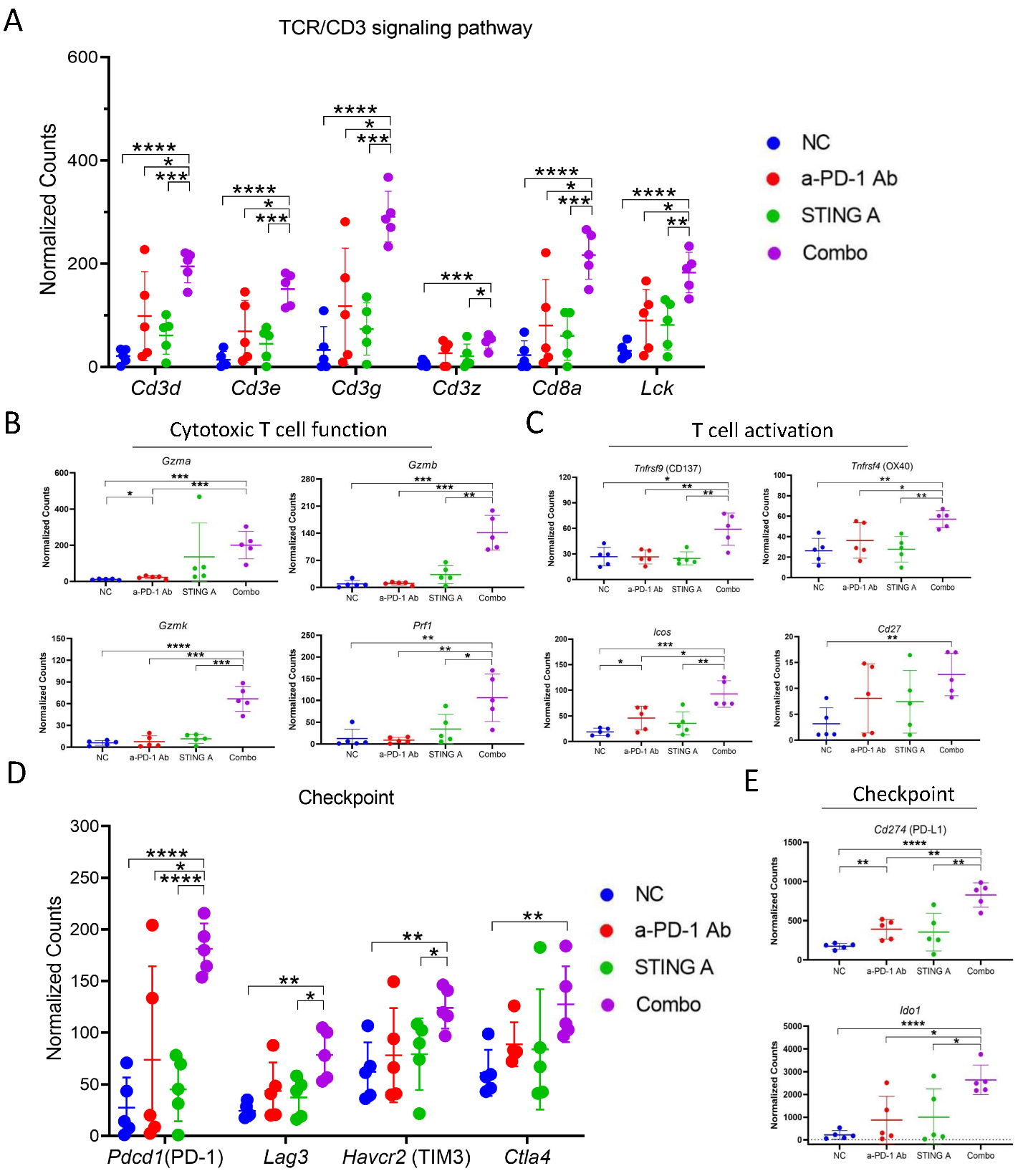


**Figure S4. Intratumoral injection of STING agonist in combination with anti-PD-1 antibody enhances T cell activation signals and reveals differential expression of effector and regulatory genes.** Among the differentially expressed genes identified in the NanoString analysis mentioned above, further statistical analysis was conducted specifically on genes associated with the activation of effector T cells. **(A)** Expression of genes in the T cell receptor (TCR)/CD3 signaling pathway in SubQ tumors from different treatment groups. As anticipated, the expression of TCR/CD3 signaling pathway genes including *Cd3d*, *Cd3e*, *Cd3g*, *Cd3z*, *Cd8a*, and *Lck* in the combo treatment group was significantly increased[22, 23]. In addition, the results revealed that *Gzma*, *Gzmb*, *Gzmk*, and *Prf1* expression was significantly elevated in the combo treatment group comparing to most of other treatment groups, suggesting that the cytotoxic function of effector T cells are significantly enhanced **(B)**. The gene expression of T cell co-stimulatory factors including *Tnfrsf9* (CD137), *Tnfrsf4* (OX40), and *Icos* were significantly increased in the combo treatment group when compared to any other group and including *Cd27* when compared to the vehicle treatment group **(C)**. However, the gene expression of co-inhibitory receptors **(D)** including *Pdcd1* (PD-1), *Lag3*, *Havcr2* (TIM3), and *Ctla4* and the expression of immune checkpoint activators **(E)** such as *Cd274* (PD-L1) and *Ido1* were significantly elevated in the combo treatment group compared to any other treatment group. These results suggested that T cell activation in the combo treatment group may also lead to the T cell exhaustion, in consistence with previously published studies[24]. NC, vehicle/isotype antibody control; STING A, STING agonist; a-PD-1 Ab, anti-PD-1 antibody; Combo, STING A+a-PD-1 Ab. Data are shown as the mean ± SD; comparison by unpaired t test; *p < 0.05; **p < 0.01; ***p < 0.001; ****p < 0.0001.


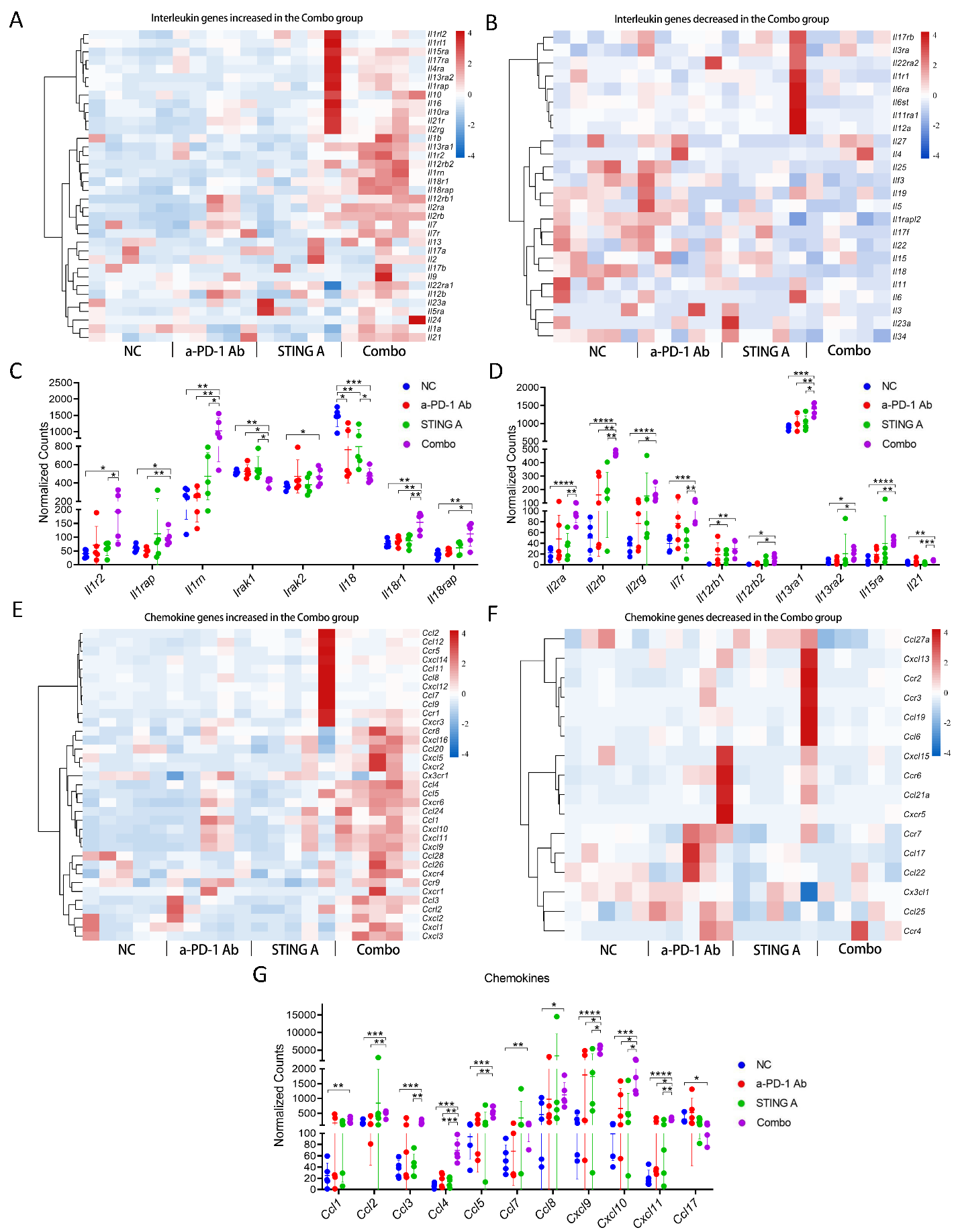


**Figure S5. Differential expression analysis of cytokines and chemokines in liver metastatic lesions following intratumoral STING Agonist and anti-PD-1 antibody treatment.** The expression of cytokines which are known to be involved in the development and differentiation of T and B lymphocytes were investigated. Heatmap of increased **(A)** and decreased **(B)** genes in the families of interleukin in the combo group comparing to the vehicle control treatment group are present. **(C)** Expression of genes, as indicated, in the gene families of interleukins (ILs) are presented. The expression of most genes in the IL-1 and IL-18 family except *Il18* itself and *Irak1* was significantly increased in the combo treatment group compared to most of other treatment groups. **(D)** Expression of genes, as indicated, in the gene families of proinflammatory cytokine are presented. The gene expression of most of the proinflammatory cytokine receptors including *Il2ra*, *Il2rb*, *Il2rg*, *Il7r*, *Il12rb1*, *Il12rb2*, *l13ra1*, *Il13ra2*, and *Il15ra* was significantly increased in the combo treatment group compared to most of other treatment groups. Interestingly, the cytokines themselves including *Il2*, *Il7*, *Il12*, and *Il13*, in addition to *Il21* which was significantly upregulated, were upregulated in a non-statistically significant trend in the combo treatment group compared to the control or single treatment groups as shown in Figure S4A. These results suggest that pro-inflammatory pathways including those that mediate the inflammasome are activated broadly by the combination of the STING agonist and anti-PD-1 antibody. Heatmap of increased **(E)** and decreased **(F)** genes in the families of chemokine in the combo group comparing to the vehicle control treatment group are present. As anticipated, the majority of chemokine genes were upregulated in the combo treatment group compared to the anti-PD-1 antibody treatment group, likely due to the innate immune response induced by the STING agonist. **(G)** Expression of genes, as indicated, in the gene families of chemokines. The results demonstrate a significantly increased expression of *Ccl1*, *Ccl2*, *Ccl3*, *Ccl4*, *Ccl5*, *Ccl7*, and *Ccl8* in the combo treatment group compared to the vehicle control treatment group, suggesting that stimulation of innate immune response is anticipated to induce myeloid cell infiltration. C-X-C motif chemokine ligand (CXCL) 9, CXCL10, and CXCL11 are known to bind C-X-C motif chemokine receptor (CXCR) 3 on T cells and, in response to IFN signaling to recruit memory and activated effector T cells[25]. A significant enhancement of the expression of *Cxcl9*, *Cxcl10*, and *Cxcl11* in the combo treatment group compared with the control or single treatment groups was observed (Figure S4G) although the expression of *Cxcr3* was similar among different treatment groups (Figure S3C). Interestingly, comparing to the vehicle control treatment group, the administration of anti-PD-1 antibody showed a statistically non-significant trend of increase whereas STING agonist showed a trend of decease in the expression of *Ccl17,* which encodes a T regulatory cell (Treg) chemokine. This finding is thus consistent with published studies showing that ICIs upregulate C-C motif chemokine ligand (CCL) 17 expression in tumors and increase the migration of Tregs into the TME of PDAC[26, 27]. Moreover, the results demonstrated that the combo treatment led to a significantly decreased expression of *Ccl17* (Figure S4F-G). Note that the gene expression results from the NanoString assay may be influenced by an influx of immune cells that express the genes. Therefore, an increased expression of certain immune genes may represent an increased infiltration of the relevant immune subtypes. Taken together, these results suggest that STING agonist may confer an antitumor effect by suppressing CCL17 expression or CCL17-expressing cells and thereby suppressing Treg migration into the tumor microenvironment. NC, vehicle/isotype antibody control; STING A, STING agonist; a-PD-1 Ab, anti-PD-1 antibody; Combo, STING A+a-PD-1 Ab. Data are shown as the mean ± SD; comparison by unpaired t test; *p < 0.05; **p < 0.01; ***p < 0.001; ****p < 0.0001.


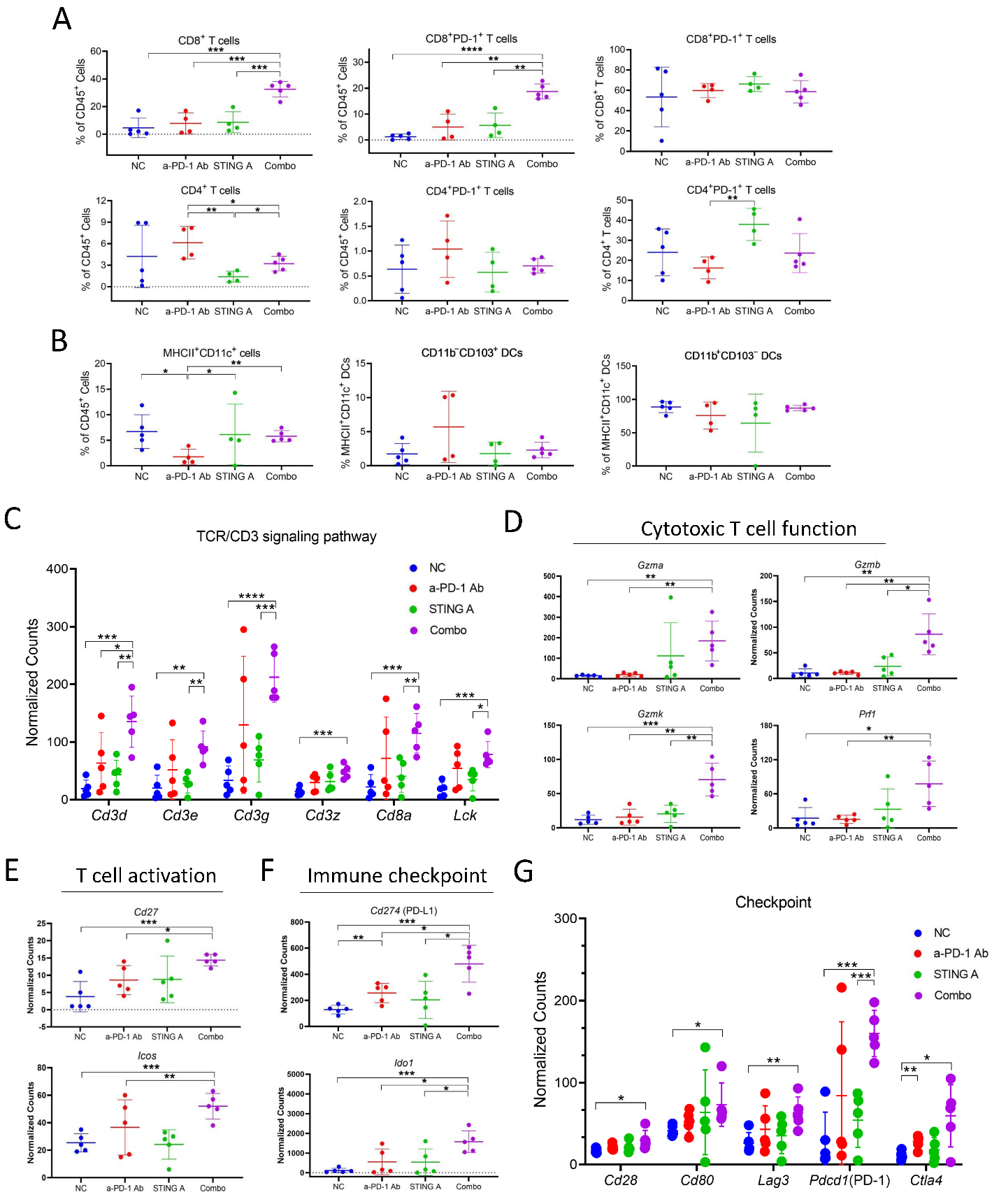


**Figure S6. Intratumoral injection of STING agonist in combination with anti-PD-1 antibody enhances effector T cell infiltration status and activation signals in distant subcutaneous tumors, mediating abscopal effects. (A)** Percentages of immune cells in the remote SubQ tumors, such as the CD8^+^, CD8^+^PD-1^+^, CD4^+^, and CD4^+^PD-1^+^ T cells among CD45^+^ leucocytes, the CD8^+^PD-1^+^ among CD8^+^ T cells, and the CD4^+^PD-1^+^ T cells among CD4^+^ T cells, respectively. The immune response that mediates the abscopal effects in the distant tumors were examined. Firstly, a significant increase in the infiltration of CD8^+^ and CD8^+^PD-1^+^ T cells in the SubQ tumors from the combo treatment group were observed, supporting the abscopal effect. Similar to the locally targeted lesions, CD4^+^ and CD4^+^PD-1^+^ T cells in the SubQ tumors were not significantly changed among all treatment groups. **(B)** Percentages of the MHCII^+^CD11c^+^ DC, CD11b^−^CD103^+^ and CD11b^+^CD103^−^ DC subtypes DC among CD45^+^ leucocytes, respectively, in the distant SubQ tumors. CD11b^−^CD103^+^ DCs in the SubQ tumors were also similar among all treatment groups, suggesting that the activation of antigen presenting cells occurred locally. Next, NanoString assay was used to assess the T cell functional status. Differentially expressed genes in the distant SubQ tumors from different treatment groups, as indicated, are presented. The results indicate that the expression of genes associated with the TCR/CD3 complex exhibited a similarly significant increase in the SubQ tumors from the combo treatment group compared to other treatment groups as in the liver metastases **(C)**. Similarly, genes related to the cytotoxic activities of effector T cells, including *Gzma*, *Gzmb*, *Gzmk*, and *Prf1*, demonstrated a significant increase in the combo treatment group compared to most of other treatment groups **(D)**. In addition, the combo treatment group exhibited a significant increase in the expression of signals related to T cell activation including *Cd27*, *Icos*, *Cd274* (PD-L1), and *Ido1* comparing to most of other treatment groups **(E-F)** and including *Cd28*, *Cd80*, *Pdcd1* (PD-1), *Lag3*, and *Ctla4* comparing to the vehicle treatment group **(G)**. NC, vehicle/isotype antibody control; STING A, STING agonist; a-PD-1 Ab, anti-PD-1 antibody; Combo, STING A+a-PD-1 Ab. Data are shown as the mean ± SD; comparison by unpaired t test; *p < 0.05; **p < 0.01; ***p < 0.001; ****p < 0.0001.


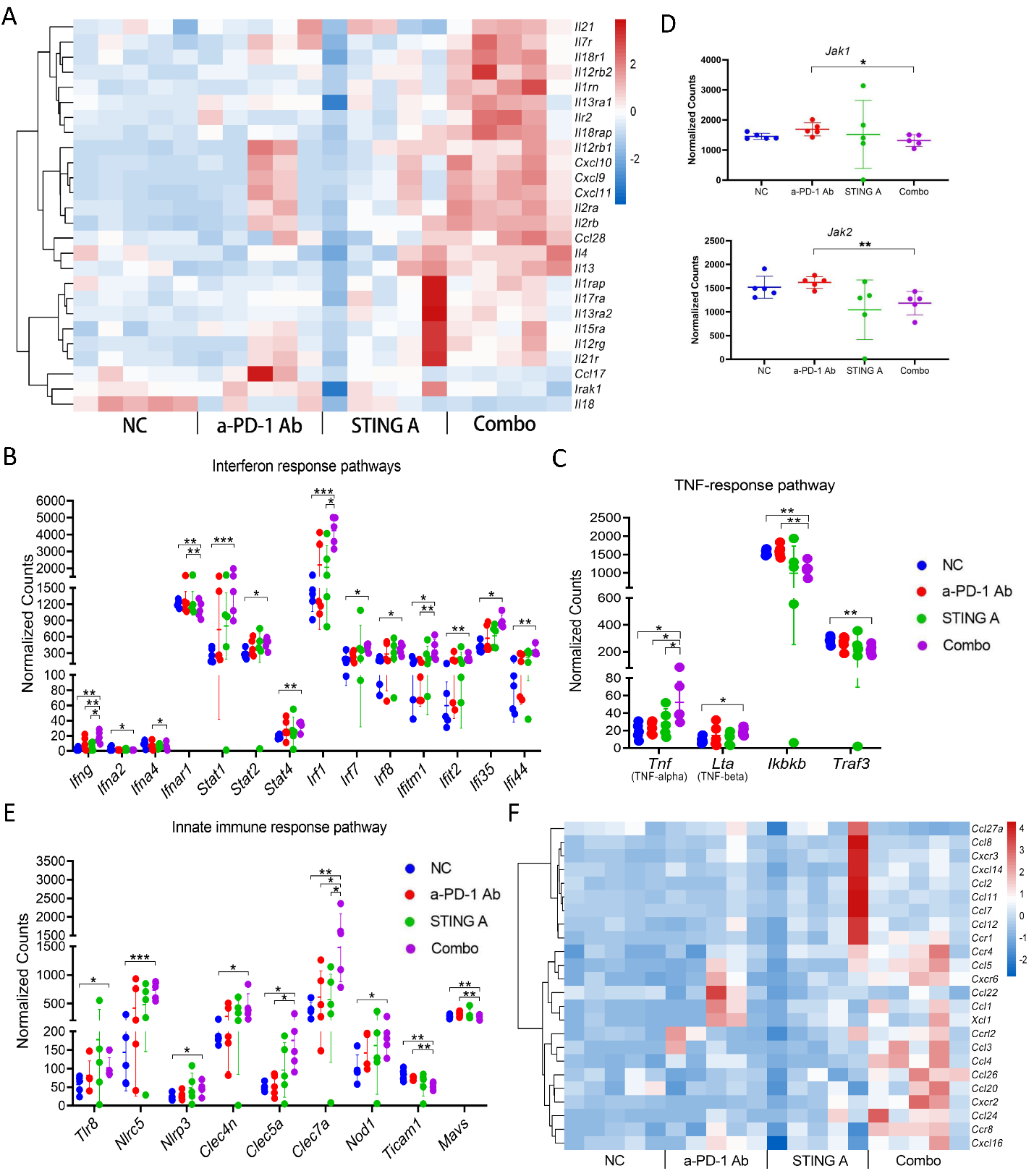


**Figure S7. Gene expression analysis reveals enhanced interleukin and chemokine signaling in distant subcutaneous tumors following intratumoral combination treatment. (A)** Heatmap of the interleukin family genes and chemokine genes that were significantly increased in the combo group comparing to the vehicle control treatment group. Consistently, genes related to chemokines for effector T cell trafficking including *Cxcl9*, *Cxcl10*, and *Cxcl11* exhibited a significant increase in their expression in the combo treatment group compared to most of other treatment groups. *Ccl17* also exhibited a significant decrease in the combo treatment group compared to the vehicle treatment group. Interestingly, the expression of *Ccl28*, a T and B cell homing factor[28], showed a significant increase in the combo treatment group in SubQ tumors, but not the target metastatic lesions (Figure S4E). The gene expression of cytokines and cytokine receptors that are relevant to the activated status of T cells was similarly increased as seen in the liver metastases, again except *Il18*, further supporting the abscopal effect. In addition, the profiles of other cytokines and chemokines in the SubQ tumors were similar to those in the targeted liver metastases and showed a significant increase in pro-inflammatory immune responses in the IFN **(B)** and TNF pathways **(C)**, but no significant changes or a decrease in *Jak1* and *Jak2* **(D)**, in the combo treatment group compared to other treatment groups. Nevertheless, expression of innate agonist receptors **(E)** and adaptors in SubQ tumors appeared to be somewhat different from that in targeted liver metastases, showing an increase in *Tlr8*, *Nlrc5*, *Nlrp3*, *Clec4n*, *Clec5a*, *Clec7a*, and *Nod1*, but a decrease in *Ticam1* and *Mavs* in the combo treatment group compared to other treatment groups. **(F)** Heatmap of the chemokine family genes that showed increasing trend in the combo group comparing to the vehicle control treatment group. However, the profile of chemokines and chemokine receptors that function in the myeloid cell trafficking was similar between targeted liver metastases and distant SubQ tumors in the combo treatment group compared to other treatment groups. NC, vehicle/isotype antibody control; STING A, STING agonist; a-PD-1 Ab, anti-PD-1 antibody; Combo, STING A+a-PD-1 Ab. Data are shown as the mean ± SD; comparison by unpaired t test; *p < 0.05; **p < 0.01; ***p < 0.001; ****p < 0.0001.


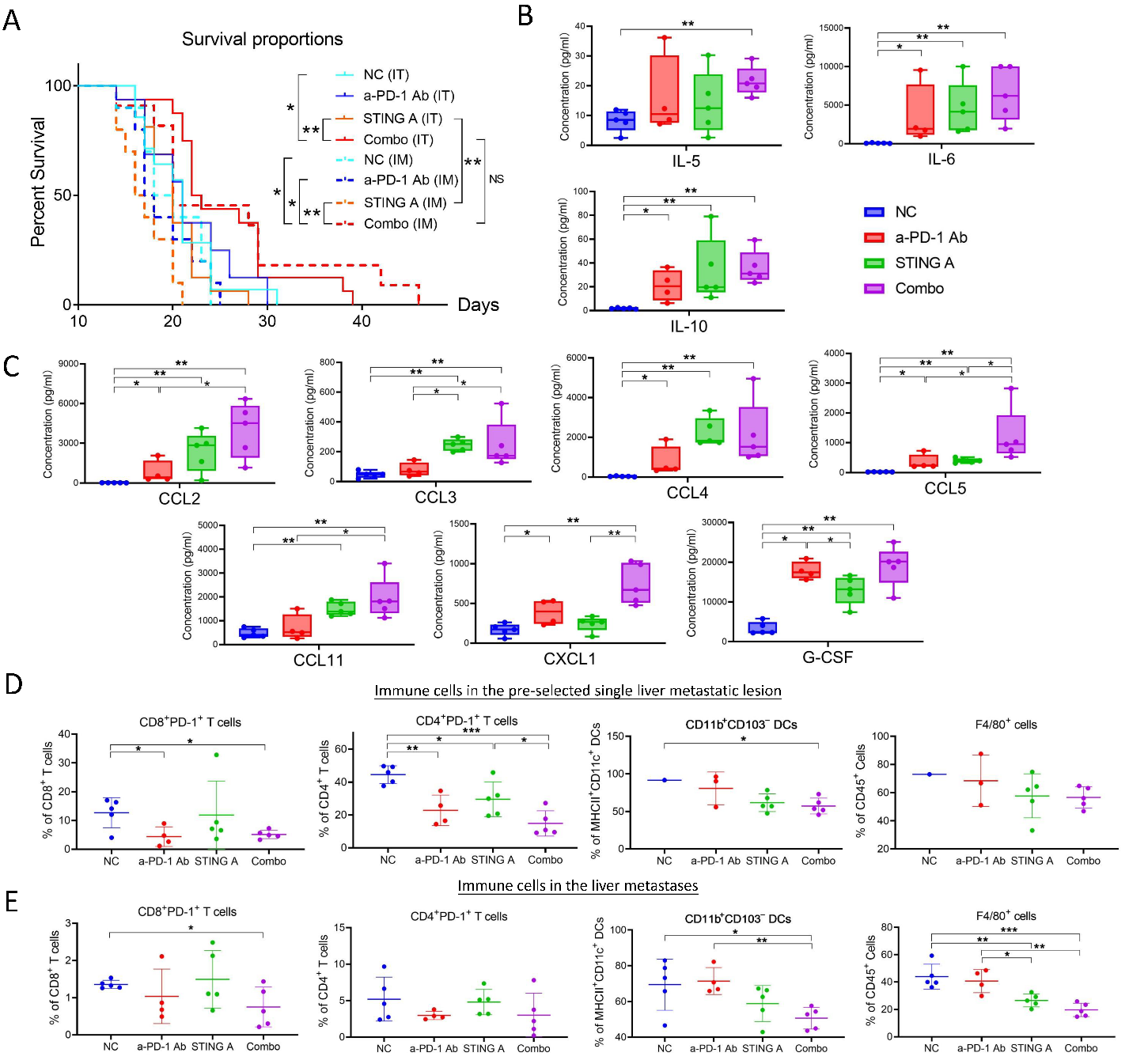


**Figure S8. Intramuscular injection of STING agonist combined with anti-PD-1 antibody induces enhanced serum cytokine levels and tumor-infiltrating immune cells.** **(A)** Combined Kaplan-Meier’s survival curves compare the survival in different intratumoral (IT) and intratumoral (IM) treatment groups. By combining the survival data in both IT and IM experiments, the results demonstrated that the majority of mice died at around 3 to 4 weeks after tumor implantation whereas those who received both STING agonist by IT or IM and anti-PD-1 antibody may live up to 6 weeks. Mice who received STING agonist intramuscularly in combination with anti-PD-1 antibody reached the longest survival beyond 6 weeks although, likely due to the small sample size, there was no significant survival difference between the IM combo and the IT combo group. **(B)** The comparison of the serologic concentration of IL-5, IL-6 and IL-10 that collected 6 hours after the first IM injection among all treatment groups. Results indicated that some interleukins including IL-5[35], IL-6[36], and IL-10[37] that support lymphocyte growth and/or antibody production were also boosted in the IM combo group. **(C)** Comparison of serum concentrations of CCL2, CCL3, CCL4, CCL5, CCL11, CXCL1, and G-CSF collected 6 hours after the first IM injection between treatment groups. The results also indicated the increased production of cytokines that participate in the recruitment of macrophages, neutrophils, and eosinophils, including CCL2, CCL3, CCL4, CCL5, CXCL1, granulocyte colony stimulating factor (G-CSF), and CCL11. Percentages of the CD8^+^PD-1^+^ among CD8^+^ T cells, the CD4^+^PD-1^+^ T cells among CD4^+^ T cells, the CD11b^+^CD103^−^ subtype DC among MHCII^+^ CD11c^+^ DCs, and F4/80^+^ cells among CD45^+^ leucocytes in the pre-selected single liver metastatic lesion **(D)** and non-target liver metastases **(E)**. NC, vehicle/isotype antibody control; STING A, STING agonist; a-PD-1 Ab, anti-PD-1 antibody; Combo, STING A+a-PD-1 Ab. Data are shown as the mean ± SD; comparison by Log-rank test for A and by unpaired t test for others; *p < 0.05; **p < 0.01; ***p < 0.001.


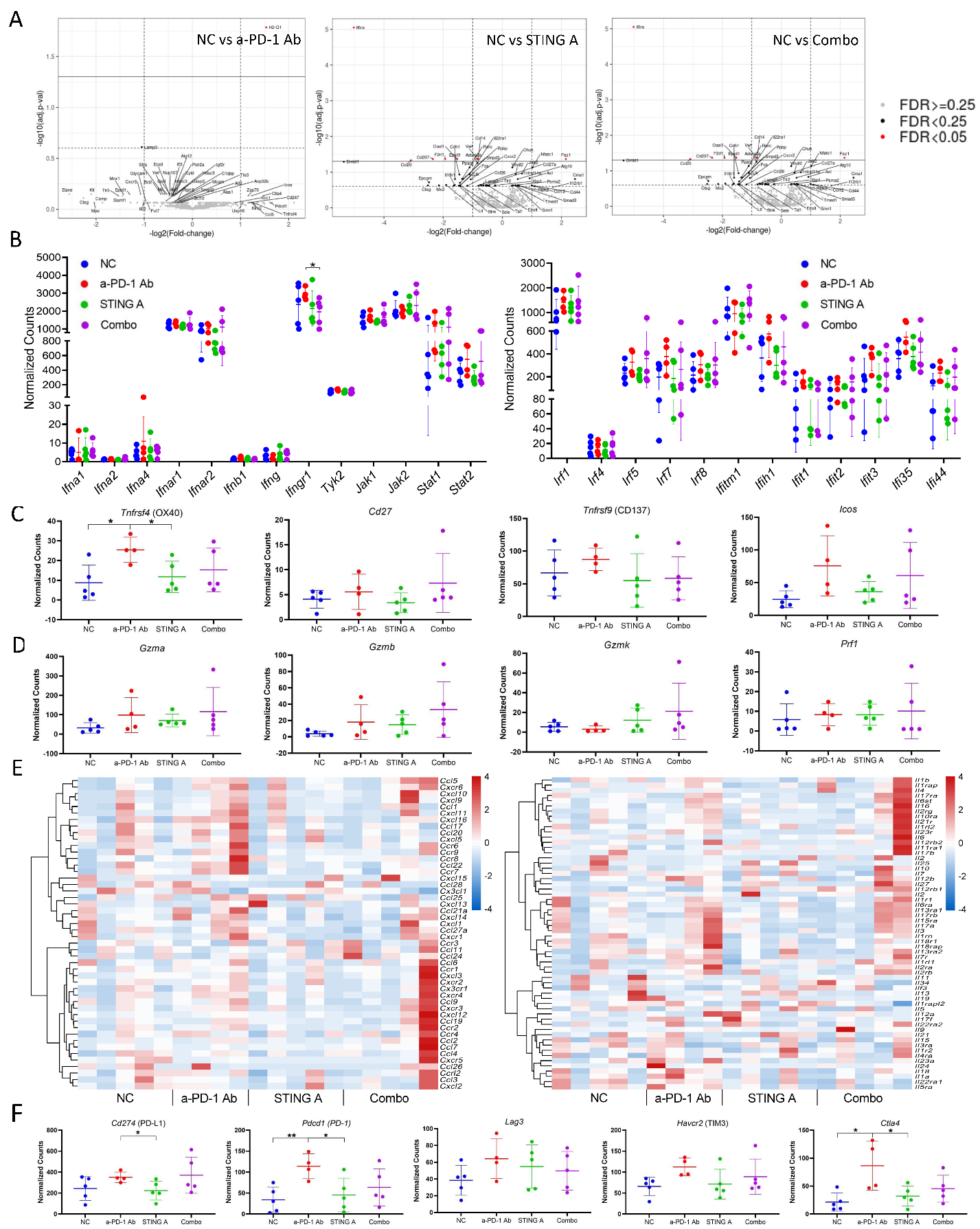


**Figure S9. Gene expression analysis of liver metastatic lesions after STING agonist systemic injection reveals moderate immune modulation but reduced immunosuppressive signals. (A)** Volcano map of differentially expressed genes in the pre-selected, target single liver metastatic lesions between the vehicle control and three treatment groups, respectively Dashed horizontal line indicates the false discovery rate at 0.25 while the solid horizontal line is at 0.05. Dashed vertical line at -1 indicates the –log_2_(Fold-change). The high-throughput analysis of the NanoString results only revealed a smaller number of genes that were differentially expressed among the treatment groups than those differentially expressed among the treatment groups with the IT injection of STING agonist. **(B)** Expression of genes in the IFN-response pathways in the pre-selected, target single liver metastatic lesions from different treatment groups. The results however supported a non-significant increasing trend in the expression of genes associated with the IFN response pathways in the liver metastases in the IM combo group. Expression of genes as indicated, in the gene families of the T cell co-stimulatory factors **(C)** and the activation of effector T cells **(D)** in the pre-selected single liver metastatic lesion. Nevertheless, in the STING agonist IM alone treatment group, the expression of T cell co-stimulatory molecules including *Tnfrsf4* (OX40), *Cd27*, *Tnfrsf9* (CD137), and *Icos* exhibited a trend consistent with the results in the single STING agonist IT treatment group (Figure S5C). However, the IM injection of STING agonist combined with anti-PD-1 antibody did not significantly increase the expression of these co-stimulatory molecules, together with the above T cell infiltration results (Figure 1E-F), suggesting anti-PD-1 antibody or anti-PD-1 antibody alone may not be an optimal immune checkpoint inhibitor treatment strategy in combination with STING agonist. Nevertheless, a strong trend of increased expression of genes related to the cytotoxic activities of effector T cells in the IM combo group were observed, including *Gzma*, *Gzmb*, *Gzmk*, and *Prf1*. **(E)** Heatmap of genes in the families of interleukin and chemokine in the pre-selected single liver metastatic lesion in the combo group comparing to the vehicle control treatment groups. Chemokines especially those involved in effector T cell trafficking including *Cxcl9*, *Cxcl10*, and *Cxcl11* exhibited similar trend as those genes associated with cytotoxic activities of effector T cells. **(F)** Expression of genes of the checkpoint gene family in pre-selected, target single liver metastatic lesions from the intramuscularly treated mice. In contrast, the increase in the T cell exhaustion and immune checkpoint signals and myeloid cell-recruiting cytokine/chemokine signals that were observed with the IT combo treatment were not observed in the IM combo group. However, an increasing trend of the expression of *Ctla-4* was observed after combinational treatment, indicating that CTLA-4 may remain one of the immunosuppressive signals induced by intramuscularly injected STING agonist. The exact mechanism warrants further investigation. NC, vehicle/isotype antibody control; STING A, STING agonist; a-PD-1 Ab, anti-PD-1 antibody; Combo, STING A+a-PD-1 Ab. Data are shown as the mean ± SD; comparison by unpaired t test; *p < 0.05.


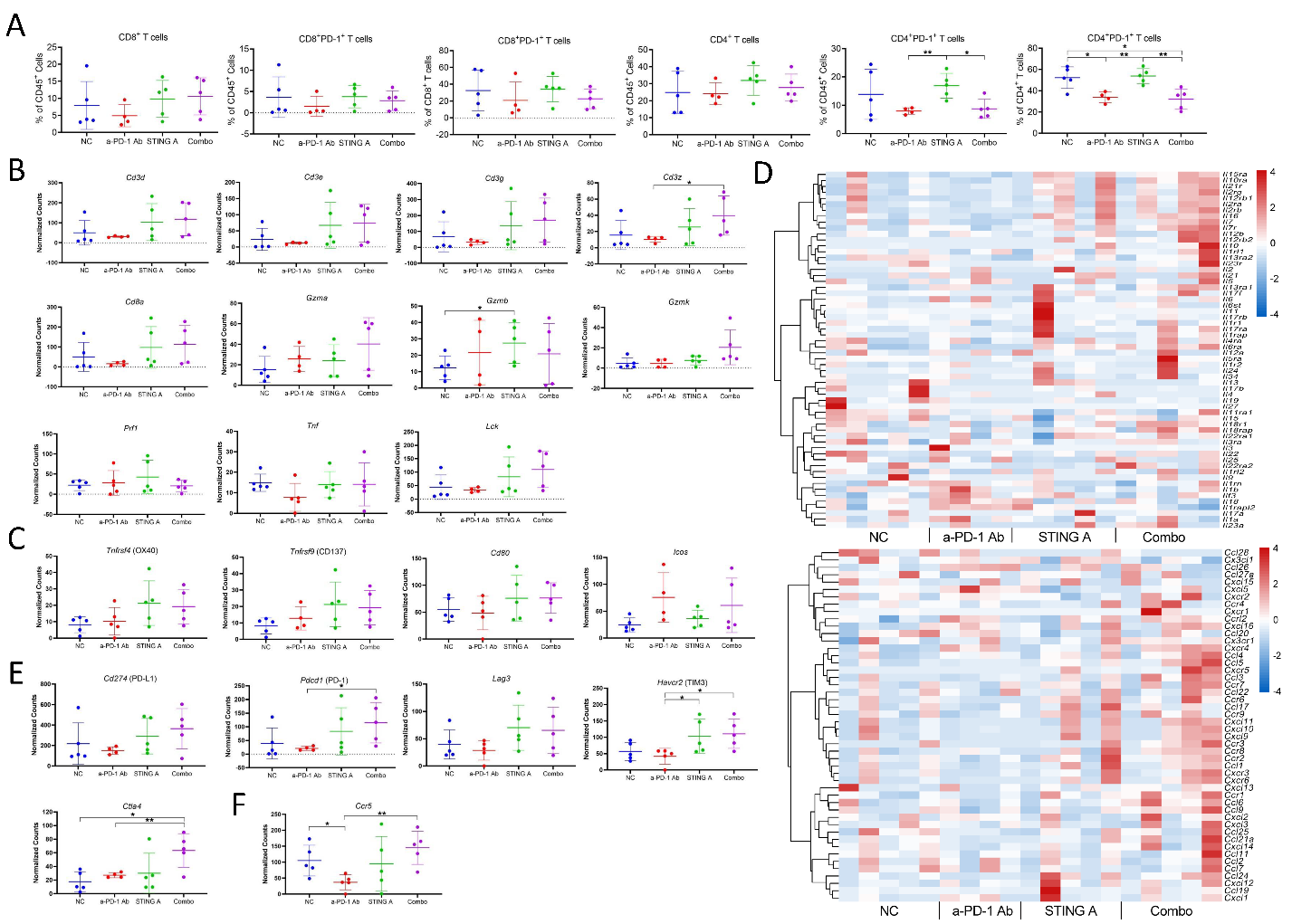


**Figure S10. Analysis of immune cell infiltration and gene expression in SubQ tumors of IM-treated mice reveals modulation of immune responses and checkpoint signaling. (A)** Percentages of immune cells in the remote SubQ tumors of IM experiment, such as the CD8^+^, CD8^+^ PD-1^+^, CD4^+^, and CD4^+^PD-1^+^ T cells among CD45^+^ leucocytes, the CD8^+^PD-1^+^ among CD8^+^ T cells, and the CD4^+^PD-1^+^ T cells among CD4^+^ T cells, respectively. The results demonstrated a significant decrease in the infiltration ratio of CD4^+^PD-1^+^ T cells in the CD4^+^ T cells in the SubQ tumors from the IM combo treatment group. A similar decreasing trend of CD8^+^PD-1^+^ T cells was observed, demonstrating the treatment effect of anti-PD-1 antibody. Next, NanoString assay was used to assess the T cell functional status within the SubQ tumors. Differentially expressed genes in the distant SubQ tumors of IM experiment from different treatment groups, including those in the gene families of T cell functional status **(B)** and T cell co-stimulatory factors **(C)**. Interestingly, unlike the results in the liver metastases, the expression of genes associated with the T-cell receptor CD3 complex exhibited a significant increase (*Cd3z*) or a strong increasing trend in the SubQ tumors from the combo treatment group compared to other treatment groups (Figure S10B). T cell co-stimulatory molecules including *Tnfrsf4* (OX40), *Tnfrsf9* (CD137), *Cd80*, and *Icos* all exhibited an increasing trend. **(D)** Heatmap of genes in the families of interleukin and chemokine in the remote SubQ tumors of IM experiment in the combo group comparing to the vehicle control treatment groups. In addition, the IM combo treatment group exhibited a significantly increased expression of *Il7r* (Figure S10D), which has been shown to play a critical role in the development of lymphocytes in the process known as V(D)J recombination[29]. These results suggest that there may be an intertumoral heterogeneity in the immune response to the systemic administration of STING agonist. **(E)** Expression of genes of the checkpoint gene family in distant subcutaneous tumors from the intramuscularly treated mice. The results indicated that the T cell exhaustion and immune checkpoint signals exhibited an enhanced expression in the IM combo treatment group compared to other treatment groups. These results suggest that an enhanced T cell activation status and cytotoxic function in response to either IT or IM treatment of STING agonist is associated with upregulation of T cell exhaustion signals and CTLA-4. **(F)** Expression of *Ccr5* in the remote SubQ tumors of IM experiment. The overall increasing trend of those chemokines/chemokine receptors involved in the myeloid cell recruitment was less significant in both liver metastases and SubQ tumors **(D)** than that in the IT combo group, suggesting that systemic delivery of STING agonist does not lead to a strong induction of immunosuppressive signals. It is noteworthy to mention that *Ccr5* expression in the SubQ tumors was significantly reduced by the treatment of anti-PD-1 antibody compared to the vehicle treatment group, but was significantly enhanced following the IM injection of STING agonist in combination with anti-PD-1 antibody. This result appears to be in consistent with the agonistic effect of CCR5 expression previously reported in the PDAC models[30]. NC, vehicle/isotype antibody control; STING A, STING agonist; a-PD-1 Ab, antiPD-1 antibody; Combo, STING A+a-PD-1 Ab. Data are shown as the mean ± SD; comparison by unpaired t test; *p < 0.05; **p < 0.01.


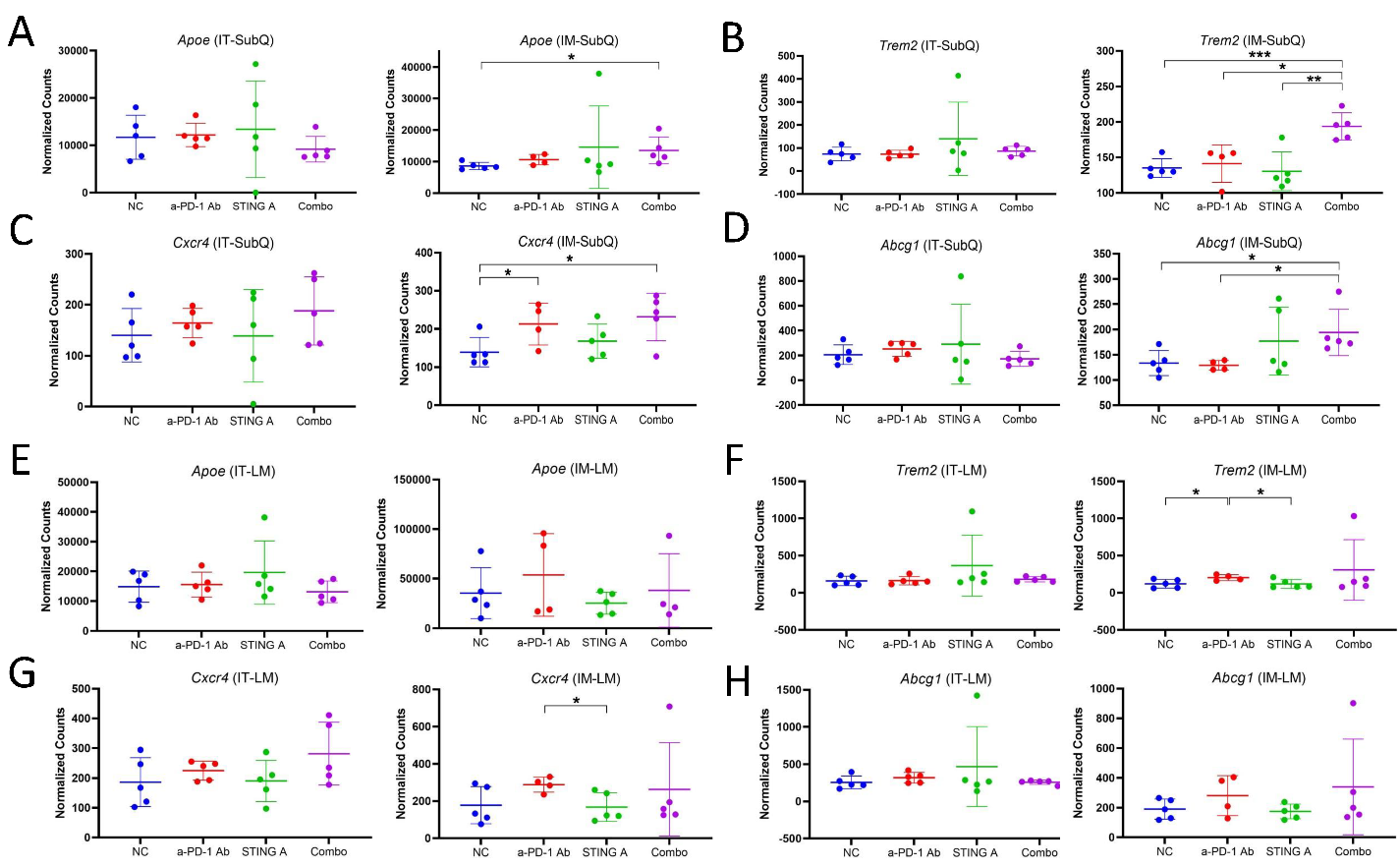


**Figure S11. Comparison of expressions of differentially expressed genes in tumors from systemically treated mice.** Expression of *Apoe*, *Trem2*, *Cxcr4*, and *Abcg1* in the distant subcutaneous tumors **(A-D)** and liver metastatic lesions **(E-H)** from both intratumoral and intramuscularly treated mice. The results revealed that the expression of immunosuppressive myeloid cells associated-genes including *Apoe*, *Trem2*, *Cxcr4*, and *Abcg1* was significantly increased in the SubQ tumors from the IM combo treatment group, but not the SubQ tumors from the IT combo treatment group, compared to the respective control group. Such a difference was not observed in the comparison between liver metastases from the IM combo treatment group and the IT combo treatment group. These results suggest that systemic administration of STING agonist could still induce certain immunosuppressive signals that require additional targeted treatments. Data shown as mean ± SD; comparison by unpaired t test; *p < 0.05; **p < 0.01; ***p < 0.001.


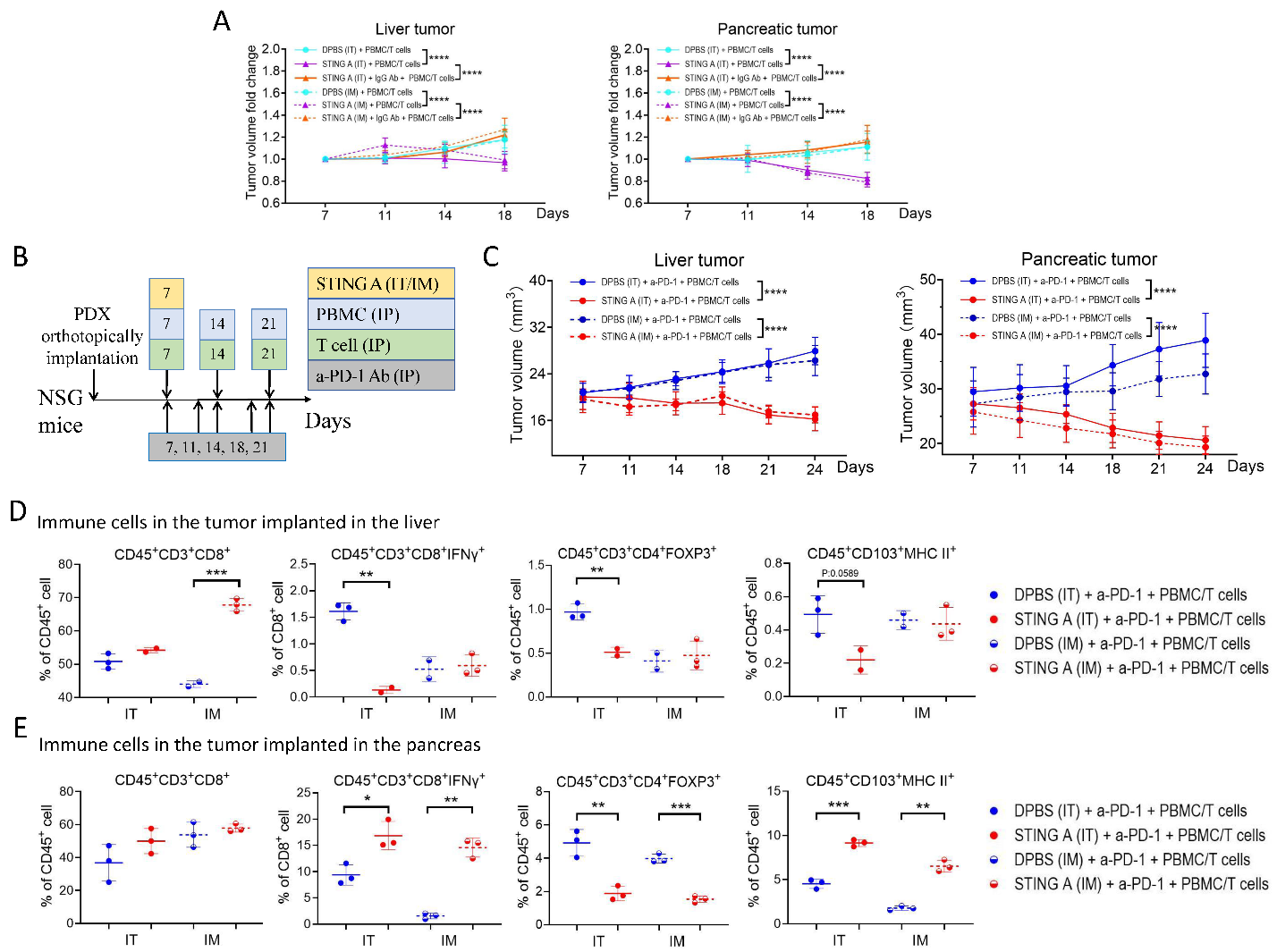


**Figure S12. Immune modulating effects of STING agonist in combination with anti-PD-1 antibody in the PDX model reconstituted with human PBMC. (A)** Comparison between the fold changes of tumor volumes before Day 18 in the control (DPBS + PBMC/T cells) and STING agonist treatment groups (STING A + PBMC/T cells) in Figure 2D and in the STING agonist treatment group (STING A + IgG Ab + PBMC/T cells) in Figure 2G. By comparing the fold changes of tumor volumes, the results demonstrated that a single injection of STING agonist with freshly thawed PBMC infused once was comparable to the DPBS control before Day 18. **(B)** Schema for the treatments of STING agonist in combination with anti-PD-1 antibody and the reconstitution of ex vivo activated T cells and freshly thawed PBMCs, as indicated, in mice implanted with human PDX used for assessing the immune modulating effects of STING agonist in combination with anti-PD-1 antibody. Both ex vivo activated T cells and freshly thawed PBMCs were infused weekly for three times. **(C)** Ultrasound measurement of tumor volumes of the orthotopic liver- and pancreas-implanted tumors treated with anti-PD-1 antibody or treated with STING agonist via IT or IM, as indicated, in combination with anti-PD-1 antibody. The results demonstrated a significant tumor suppression by the combination of STING agonist and anti-PD-1 antibody compared to anti-PD-1 antibody alone. Percentages of the CD8^+^ T cells among CD45^+^ cells, percentages of the CD8^+^ IFNγ^+^ T cells among CD8^+^ T cells, percentages of the CD4^+^ FOXP3^+^ Treg, and percentages of the CD103^+^ subtype DC among CD45^+^ cells in the tumor implanted in the liver **(D)** and pancreas **(E)**, respectively. This result demonstrated that human CD8^+^ T cell infiltration are present in all these tumors. Interestingly, IM, but not IT, injection of STING agonist leads to a significant increase of CD8^+^ T cell infiltration in the liver-implanted tumors, but not pancreas-implanted tumors. Moreover, a significant decrease in the IFNγ^+^CD8^+^ cytotoxic effector T cell subset was observed in IT-injected liver-implanted tumors and may be attributed to an increased T cell exhaustion signal as demonstrated above (Figure S11). Again, both IT and IM injection of STING agonist led to a comparable abscopal effect with an increased IFNγ^+^CD8^+^ cytotoxic effector T cell infiltration in the pancreas-implanted tumors. Interestingly, in the IT, but not IM, treatment group, a significant reduction in CD4^+^FOXP3^+^ Tregs was observed. In addition, IT, but not IM, administration of STING agonist in combination with anti-PD-1 antibody, comparing anti-PD-1 antibody alone, resulted in a significant decrease in DC infiltration in the liver-implanted tumors although CD103^+^ DC infiltration was significantly increased in the pancreas-implanted tumors. STING A, STING agonist; a-PD-1 Ab, anti-PD-1 antibody; PBMC, peripheral blood mononuclear cell; PDX, patient-derived xenograft; IT, intratumoral; IM, intramuscular; IP, intra-peritoneal. Data shown as mean ± SD; comparison by two-way ANOVA and Welch’s t test; *p < 0.05; **p < 0.01; ***p < 0.001; ****p < 0.0001.


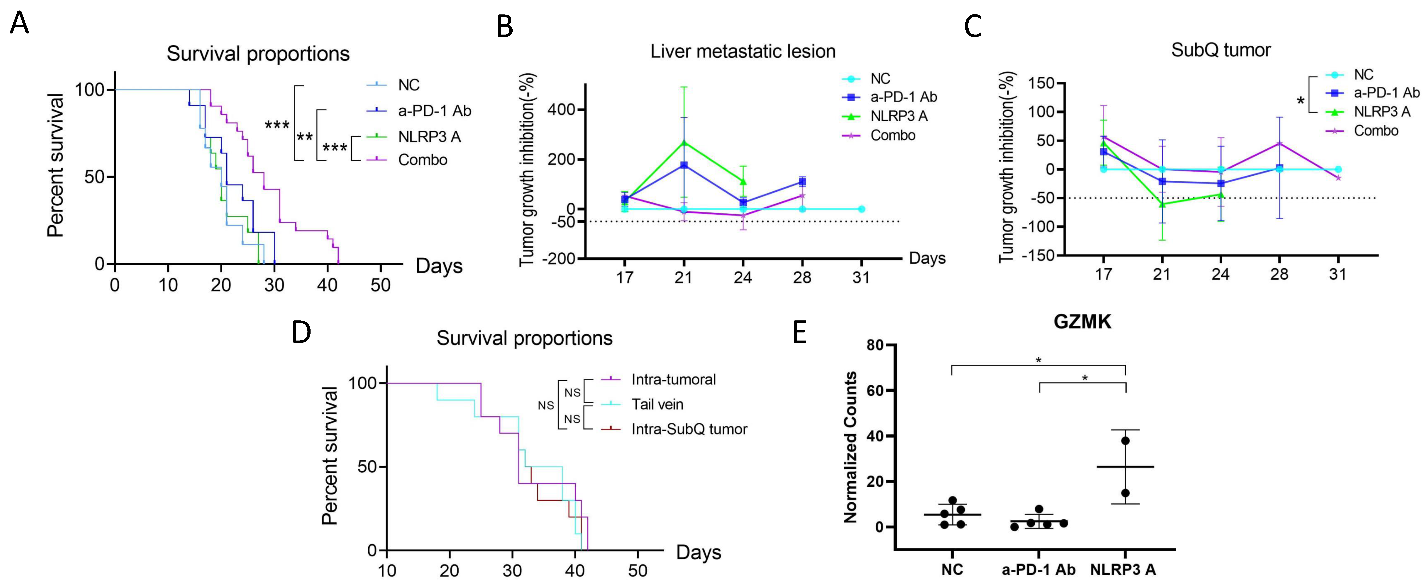


**Figure S13. Systemic administration and intratumoral administration of NLRP3 agonist in combination with anti-PD-1 antibody induced antitumor efficacy. (A)** Kaplan-Meier’s survival curves compare different treatment groups. The combination of IT injection of NLRP3 agonist and anti-PD-1 antibody had similar antitumor efficacy as the combination of IT injection of STING agonist and anti-PD-1 antibody and prolonged the survival of liver metastasis mice implanted with SubQ tumors as compared to anti-PD-1 antibody alone, NLRP3 agonist alone, or vehicle control. TGI of the injected liver metastatic lesion **(B)** and remote SubQ tumor **(C)** during the treatment period. Dashed line at -50% indicates statistically significant TGI. Likely due to the small sample size, the combination of NLRP3 agonist and anti-PD-1 antibody failed to induce a significantly stronger tumor growth inhibition on either liver metastatic lesion or the SubQ tumors than single treatment groups. **(D)** Kaplan-Meier’s survival curves comparing different routes of administration of NLRP3 agonist including intratumoral, tail-vein injection, and intra-subcutaneous tumor injection. Different routes of administration of NLRP3 agonist including IT, tail-vein injection, or intra-subcutaneous tumor injection resulted in no survival difference of the liver metastasis mice. **(E)** Expression of *Gzmk* in the target liver metastatic lesions from different treatment groups. Despite a small sample size, NanoString analysis showed that the expression of *Gzmk,* an effector T cell cytotoxicity-associated gene, was significantly increased in the NLRP3 agonist-treated tumor, but not in the STING agonist-treated tumor (Figure S5B). Although it may have been limited by the small sample size, this study demonstrated that there were no significant differences in anti-tumor efficacy between intratumoral and systemic administration of NLRP3 agonist. This study has not performed an in-depth investigation on the NLRP3 agonist. However, with limited data, this study suggests that further investigation of systemic administration of other innate immune agonists such as NLRP3 agonist is warranted. NC, vehicle/isotype antibody control; NLRP3 A, NLRP3 agonist; a-PD-1 Ab, anti-PD-1 antibody; Combo, NLRP3 A+a-PD-1 Ab. Data are shown as the mean ± SD; comparison by Log-rank test for A and D, and by unpaired t test for others; *p < 0.05; **p < 0.01; ***p < 0.001; NS, not significant.

References

1. Hingorani SR, Wang L, Multani AS, Combs C, Deramaudt TB, Hruban RH, et al. Trp53R172H and KrasG12D cooperate to promote chromosomal instability and widely metastatic pancreatic ductal adenocarcinoma in mice. Cancer cell. 2005;7(5):469-83.

2. Soares KC, Foley K, Olino K, Leubner A, Mayo SC, Jain A, et al. A preclinical murine model of hepatic metastases. J Vis Exp. 2014(91):51677.

3. Barber GN. STING: infection, inflammation and cancer. Nature Reviews Immunology. 2015;15(12):760.

4. Zhang T, Ma C, Zhang Z, Zhang H, Hu H. NF‐κB signaling in inflammation and cancer. MedComm. 2021;2(4):618-53.

5. Kwon J, Bakhoum SF. The cytosolic DNA-sensing cGAS–STING pathway in cancer. Cancer discovery. 2020;10(1):26-39.

6. Shadab A, Mahjoor M, Abbasi-Kolli M, Afkhami H, Moeinian P, Safdarian A-R. Divergent functions of NLRP3 inflammasomes in cancer: a review. Cell Communication and Signaling. 2023;21(1):232.

7. Ni H, Zhang H, Li L, Huang H, Guo H, Zhang L, et al. T cell-intrinsic STING signaling promotes regulatory T cell induction and immunosuppression by upregulating FOXP3 transcription in cervical cancer. Journal for Immunotherapy of Cancer. 2022;10(9).

8. Amouzegar A, Chelvanambi M, Filderman JN, Storkus WJ, Luke JJ. STING agonists as cancer therapeutics. Cancers (Basel). 2021;13(11):2695.

9. Le Naour J, Zitvogel L, Galluzzi L, Vacchelli E, Kroemer G. Trial watch: STING agonists in cancer therapy. Oncoimmunology. 2020;9(1):1777624.

10. Ding C, Song Z, Shen A, Chen T, Zhang A. Small molecules targeting the innate immune cGAS‒STING‒TBK1 signaling pathway. Acta Pharmaceutica Sinica B. 2020;10(12):2272-98.

11. Schieven G, Brown J, Swanson J, Stromko B, Ho C, Zhang R, et al., editors. Preclinical characterization of BMS-986301, a differentiated STING agonist with robust antitumor activity as monotherapy or in combination with anti-PD-1. Proceedings of the 33rd Annual Meeting & Pre-Conference Programs of the Society for Immunotherapy of Cancer (SITC 2018), Washington, DC, USA; 2018.

12. Vonderhaar EP, Barnekow NS, McAllister D, McOlash L, Eid MA, Riese MJ, et al. STING activated tumor-intrinsic type I interferon signaling promotes CXCR3 dependent antitumor immunity in pancreatic cancer. Cellular and molecular gastroenterology and hepatology. 2021;12(1):41-58.

13. Bi C, Chadwick J, Davies ML, DelMonte AJ, Geng P, Glace AW, et al. Coupling-Condensation Strategy for the Convergent Synthesis of an Imidazole-Fused 2-Aminoquinoline NLRP3 Agonist. The Journal of Organic Chemistry. 2022;88(1):384-94.

14. Wang J, Zhang T, Li P, Gai J, Chen S, Espinoza G, et al. Engineered TCR T-cell therapy targeting mass spectrometry-identified natural epitope in PDAC. Cancer Lett. 2023;573:216366.

15. Li P, Rozich N, Wang J, Wang J, Xu Y, Herbst B, et al. Anti-IL-8 antibody activates myeloid cells and potentiates the anti-tumor activity of anti-PD-1 antibody in the humanized pancreatic cancer murine model. Cancer Lett. 2022;539:215722.

16. Tanaka Y, Chen ZJ. STING specifies IRF3 phosphorylation by TBK1 in the cytosolic DNA signaling pathway. Sci Signal. 2012;5(214):ra20.

17. Le DT, Huynh TR, Burt B, Van Buren G, Abeynaike SA, Zalfa C, et al. Natural killer cells and cytotoxic T lymphocytes are required to clear solid tumor in a patient-derived xenograft. JCI Insight. 2021;6(13).

18. Lin S, Huang G, Cheng L, Li Z, Xiao Y, Deng Q, et al. Establishment of peripheral blood mononuclear cell-derived humanized lung cancer mouse models for studying efficacy of PD-L1/PD-1 targeted immunotherapy. mAbs. 2018;10(8):1301-11.

19. Blair AB, Kleponis J, Thomas DL, 2nd, Muth ST, Murphy AG, Kim V, et al. IDO1 inhibition potentiates vaccine-induced immunity against pancreatic adenocarcinoma. J Clin Invest. 2019;129(4):1742-55.

20. Tang H, Panemangalore R, Yarde M, Zhang L, Cvijic ME. 384-well multiplexed luminex cytokine assays for lead optimization. Journal of biomolecular screening. 2016;21(6):548-55.

21. Joffre OP, Segura E, Savina A, Amigorena S. Cross-presentation by dendritic cells. Nature Reviews Immunology. 2012;12(8):557-69.

22. Guy CS, Vignali KM, Temirov J, Bettini ML, Overacre AE, Smeltzer M, et al. Distinct TCR signaling pathways drive proliferation and cytokine production in T cells. Nature immunology. 2013;14(3):262-70.

23. Lipp AM, Juhasz K, Paar C, Ogris C, Eckerstorfer P, Thuenauer R, et al. Lck mediates signal transmission from CD59 to the TCR/CD3 pathway in Jurkat T cells. PLoS One. 2014;9(1):e85934.

24. Wherry EJ, Kurachi M. Molecular and cellular insights into T cell exhaustion. Nature Reviews Immunology. 2015;15(8):486-99.

25. Tokunaga R, Zhang W, Naseem M, Puccini A, Berger MD, Soni S, et al. CXCL9, CXCL10, CXCL11/CXCR3 axis for immune activation–a target for novel cancer therapy. Cancer treatment reviews. 2018;63:40-7.

26. Mizukami Y, Kono K, Kawaguchi Y, Akaike H, Kamimura K, Sugai H, et al. CCL17 and CCL22 chemokines within tumor microenvironment are related to accumulation of Foxp3+ regulatory T cells in gastric cancer. Int J Cancer. 2008;122(10):2286-93.

27. Marshall LA, Marubayashi S, Jorapur A, Jacobson S, Zibinsky M, Robles O, et al. Tumors establish resistance to immunotherapy by regulating Treg recruitment via CCR4. Journal for immunotherapy of cancer. 2020;8(2).

28. Mohan T, Deng L, Wang B-Z. CCL28 chemokine: an anchoring point bridging innate and adaptive immunity. Int Immunopharmacol. 2017;51:165-70.

29. Baizan-Edge A, Stubbs BA, Stubbington MJ, Bolland DJ, Tabbada K, Andrews S, et al. IL-7R signaling activates widespread VH and DH gene usage to drive antibody diversity in bone marrow B cells. Cell Reports. 2021;36(2):109349.

30. Wang J, Saung MT, Li K, Fu J, Fujiwara K, Niu N, et al. CCR2/CCR5 inhibitor permits the radiation-induced effector T cell infiltration in pancreatic adenocarcinoma. The Journal of experimental medicine. 2022;219(5).
